# Effect of *EGLN1* Genetic Polymorphisms on Hemoglobin Concentration in Andean Highlanders

**DOI:** 10.1155/2020/3436581

**Published:** 2020-11-15

**Authors:** Yoshiki Yasukochi, Takayuki Nishimura, Juan Ugarte, Mayumi Ohnishi, Mika Nishihara, Guillermo Alvarez, Hideki Fukuda, Victor Mendoza, Kiyoshi Aoyagi

**Affiliations:** ^1^Department of Human Functional Genomics, Advanced Science Research Promotion Center, Mie University, Tsu, Mie 514-8507, Japan; ^2^Department of Public Health, Nagasaki University Graduate School of Biomedical Sciences, 1-12-4 Sakamoto, Nagasaki 852-8523, Japan; ^3^Department of Human Science, Faculty of Design, Kyushu University, 4-9-1 Shiobaru, Minamiku, Fukuoka 815-8540, Japan; ^4^Faculty of Dentistry, Universidad Mayor de San Andres, Av. Saavedra 2244, La Paz, Bolivia; ^5^Department of Health Sciences, Nagasaki University Graduate School of Biomedical Sciences, 1-12-4 Sakamoto, Nagasaki 852-8523, Japan; ^6^National Institute of Public Health, Japan 2-3-6 Minami, Wako City, Saitama 351-0197, Japan

## Abstract

The physiological characteristics of Andean natives living at high altitudes have been investigated extensively, with many studies reporting that Andean highlanders have a higher hemoglobin (Hb) concentration than other highlander populations. It has previously been reported that positive natural selection has acted independently on the egl-9 family hypoxia inducible factor 1 (*EGLN1*) gene in Tibetan and Andean highlanders and is related to Hb concentration in Tibetans. However, no study has yet revealed the genetic determinants of Hb concentration in Andeans even though several single-nucleotide polymorphisms (SNPs) in *EGLN1* have previously been examined. Therefore, we explored the relationship between hematological measurements and tag SNPs designed to cover the whole *EGLN1* genomic region in Andean highlanders living in Bolivia. Our findings indicated that haplotype frequencies estimated from the *EGLN1* SNPs were significantly correlated with Hb concentration in the Bolivian highlanders. Moreover, we found that an Andean-dominant haplotype related to high Hb level may have expanded rapidly in ancestral Andean highlander populations. Analysis of genotype data in an ~436.3 kb genomic region containing *EGLN1* using public databases indicated that the population structure based on *EGLN1* genetic markers in Andean highlanders was largely different from that in other human populations. This finding may be related to an intrinsic or adaptive physiological characteristic of Andean highlanders. In conclusion, the high Hb concentrations in Andean highlanders can be partly characterized by *EGLN1* genetic variants.

## 1. Introduction

Physiological phenotypes of the ancestors of modern humans adapted to various new environments across the world through genetic changes affecting their adaptive phenotypes [1–5]. High-altitude environments are extreme due to hypobaric hypoxia conditions. To understand human tolerance to such severe conditions, many studies have investigated the intrinsic physiological traits of modern humans who have settled at high altitudes, particularly Andean, Ethiopian, and Tibetan populations [6–12]. It has been reported that the physiological response to hypobaric hypoxia stress varies among these three high-altitude populations [11–14]. For instance, Andeans have a relatively high hemoglobin (Hb) concentration; thus, high Hb levels in Andeans may help them adapt to high-altitude environments. Conversely, Tibetan highlanders have a moderate Hb concentration that is comparable to that of individuals residing at sea-level because of increased plasma volume, and this may be an adaptive advantage in high-altitude environments as it results in a lower blood viscosity promoting blood flow and oxygen delivery, protection against polycythemia due to elevated red blood cell volume, and enhanced exercise capacity [11, 12, 15–17].

In recent years, several genes in the hypoxia-inducible factor (HIF) pathway have been identified as genetic factors responsible for the moderate Hb concentration in Tibetan highlanders [15, 16, 18–21]. Although previous studies have reported candidate genes involved in high-altitude adaptation in Andean populations [22–26], none have yet revealed the genes responsible for the relatively high Hb concentration in Andean highlanders. Meanwhile, recent genome-wide scans have independently detected signals of positive natural selection in genomic regions around the egl-9 family hypoxia inducible factor 1 (*EGLN1*) gene in Andean (Peruvian) and Tibetan highlanders [26]. *EGLN1* encodes the prolyl hydroxylase domain 2 (PHD2) enzyme, which is a cellular oxygen sensor protein that plays a role in the HIF pathway by regulating HIF expression levels, resulting in altered oxygen homeostasis, erythropoiesis, and angiogenesis [27, 28]. A previous study detected two missense mutations in exon 1 of *EGLN1* (rs186996510 and rs12097901) in Tibetans residing in Virginia and Utah (the USA); these mutations may enhance the degradation of HIF under hypoxic conditions, resulting in the inhibition of the HIF-mediated proliferation of erythropoiesis [19]. Moreover, the authors reported that positive natural selection may have acted on the *EGLN1* genomic region because the population differentiation analysis showed extreme allele frequency differentiation at several *EGLN1* single-nucleotide polymorphisms (SNPs) between Tibetan and Mongolian or European populations. Of note, this appeared to be attributed to genetic hitchhiking with the missense mutation at rs186996510. In addition, the allelic divergence of rs186996510 between Tibetans and Han Chinese was remarkable across the entire *EGLN1* genomic region and a signal of positive selection was detected in this genomic region [29]. The study also showed that the association between rs186996510 and Hb concentration in Tibetans was statistically significant. In Tibetans residing at different altitudes and geographic locations in China and India, the frequency of the *EGLN1* haplotype comprising the missense mutations of rs186996510 and rs12097901 was positively correlated with altitude [30], suggesting that the haplotype could be adaptive to high-altitude environments. A genome-wide sequence variation analysis in seven Tibetan populations across the Himalayan region in China also identified a signal of positive natural selection in the *EGLN1* genomic region [31]. Furthermore, the frequency of the *EGLN1* rs480902-T allele that was a putative protective allele for high-altitude pulmonary edema (HAPE) was positively correlated with altitude across the world [32, 33].

Previous studies have also reported the association between *EGLN1* SNPs and blood oxygen saturation levels in lowlander populations. In Indian lowlanders, several SNPs including rs480902 in *EGLN1* were associated with the prevalence of HAPE and with the arterial oxygen saturation (SaO_2_) levels [34]. The association between rs480902 and the levels of SaO_2_ was also observed in lowland Han Chinese patients with acute mountain sickness [35]. Further, an acute hypobaric hypoxic experiment in the context of Japanese lowlanders allowed the association of two *EGLN1* SNPs (rs12097901 and rs2790859) with the percutaneous arterial oxygen saturation (SpO_2_) response. However, the putative adaptive alleles in highlander populations appeared to be maladaptive to the acute hypobaric hypoxic exposure in the Japanese cohort, likely due to the difference of genetic backgrounds between the ethnic groups [36]. In addition, the interaction of *EGLN1* SNPs and the endothelial PAS domain protein 1 (*EPAS1*) gene may affect the prevalence of HAPE in Japanese individuals [37]. Consequently, *EGLN1* is likely involved in the regulation of the cardiovascular system and in the adaptation to high-altitudes.

It has been reported that rs1769792 showed the strongest signal of positive selection in the *EGLN1* genomic region in the Andeans [26]. Moreover, SNPs in *EGLN1* have been associated with Hb concentration in Tibetan highlanders [38]; therefore, this gene may be involved in controlling Hb concentration in Andean highlanders. However, to the best of our knowledge, there is no evidence suggesting that *EGLN1* SNPs affect the Hb dynamics of Andean highlanders. For instance, a previous study showed no significant association between five *EGLN1* SNPs and Hb levels in Andean highlanders [39]. This might be owing to the limited number of SNPs examined. Therefore, we explored the relationships between physiological measurements related to the cardiovascular system and several tag SNPs covering the entire genomic region of *EGLN1* in Andean highlanders living in Bolivia.

## 2. Materials and Methods

### 2.1. Study Subjects

A total of 99 high-altitude dwelling Andeans with Aymara or Quechua ancestry (49 men and 50 women) aged ≥20 years of age (meanage ± standarddeviation, 25.1 ± 3.2years) from two universities in Bolivia located at altitudes of ~3700 m (Universidad Mayor de San Andrés, 24 men and 24 women) and ~4000 m (Public University of El Alto, 25 men and 26 women) participated in this study. The study subjects had no documented clinical problems such as cardiovascular and respiratory diseases. Of note, these subjects were also recruited in our previous studies on toothache experience and physiological traits [40, 41].

### 2.2. Physiological Data Collection

Physiological data (height, weight, and body mass index (BMI), Hb concentration, SpO_2_, heart rate, and systolic (SBP) and diastolic (DBP) blood pressure) were acquired from the study subjects in December 2016 during health checkups at a room temperature of 23–25°C. SpO_2_ and Hb concentration measurements were obtained using a pulse oximeter (Masimo Radical V 5.0, Masimo Corp., Irvine, CA, USA) and an ASTRIM FIT Health Monitoring Analyzer (Sysmex, Kobe, Japan), respectively, after these noninvasive-measurement devices were attached to the fingertips of subjects that had rested in the sitting position. Since the measurements are estimated values, they were used to examine the magnitude relationships among subjects. SBP, DBP, and heart rate were measured in the sitting position using a digital automatic blood pressure monitor (OMRON HEM-7210, Kyoto, Japan). Interviews were conducted to obtain lifestyle information (current smoking, exercise, and drinking status).

### 2.3. DNA Extraction and SNP Genotyping

Saliva was collected from the subjects using an Oragene® DNA kit (DNA Genotek, Ottawa, Canada), and genomic DNA was purified using the prepIT®.L2P reagent (DNA Genotek) according to the manufacturer's protocol. Three SNPs (rs2486740, rs508618, and rs1769792) around *EGLN1* were selected as tag SNPs (*r*^2^ threshold: 0.75; minimum minor allele frequency: 0.3; Hardy-Weinberg equilibrium (HWE) *p* value cutoff: 0.01) using Haploview ver. 4.2 [42] with the variant call format (VCF) data of the PEL (Peruvian in Lima, Peru) population from the 1000 Genomes Project (1KGP) database (http://www.internationalgenome.org/ [43]). The SNPs were genotyped using a TaqMan SNP Genotyping Assay (Applied Biosystems, CA, USA). In addition, the SNPs rs186996510 and rs12097901, located in exon 1 of *EGLN1*, were genotyped by PCR-direct sequencing as they may have undergone positive selection in Tibetan highlanders [19, 29]. PCR amplification and direct sequencing were performed as described previously [36].

Of the five SNPs examined, rs186996510 was removed from further analysis as it was monomorphic in the Bolivian cohort. The remaining four SNPs covering the whole *EGLN1* genomic region were used to examine their genotype frequencies. The SNP genotype data for each individual are shown in Supplement Table 1. Significant deviations from the HWE for these SNPs were tested using Fisher's exact test in PLINK 1.90 (http://pngu.mgh.harvard.edu/purcell/plink/ [44]).

### 2.4. Relationships between Gene Expression and SNPs

The distribution of *EGLN1* gene expression in different human tissues and organs was investigated using the Genotype-Tissue Expression (GTEx) Portal (https://gtexportal.org/ [45]) and The Human Protein Atlas (http://www.proteinatlas.org/ [46, 47]) databases. To examine the relationships between the focal SNPs and *EGLN1* expression, we searched the results of expression quantitative trait locus (eQTL) analysis in the GTEx Portal database. A *p* value of <0.00014, as determined using the GTEx eQTL Calculator, was considered statistically significant. The effects of nucleotide substitution on protein function were predicted using the Combined Annotation Dependent Depletion (CADD) [48] scores.

### 2.5. Statistical Analyses

The genotype data for all subjects were converted into numeric data with dominant and additive genetic models. The dominant model was defined as “0, AA; 1, AB + BB” (A: major allele; B: minor allele), whereas the additive model was defined as “0, AA; 1, AB; 2, BB.” The significance of differences in the physiological measurements between subjects with different genotype groups defined by the dominant model was assessed using Welch's *t*-test. Linear regression analysis was performed to examine correlations between the focal SNPs and several hematological parameters in the additive model. Based on Bonferroni's correction, a *p* value of <0.0125 (0.05/4 SNPs examined) was considered statistically significant for the association study.

Generalized linear models (GLMs) were used to assess the relationships between Hb concentration and anthropometric phenotypes (height, weight, and BMI) or the lifestyle factors in the additive genetic model, with a conventional *p* value threshold of 0.05. Since GLM showed no correlation between Hb levels and the anthropometric phenotypes or lifestyle factors, we tested the association between genotypes and Hb levels using GLM analysis after adjusting for potential covariates (age, sex, and altitude) without the anthropometric or lifestyle parameters. A Gaussian distribution was selected for the family with an identity link in the GLM model because Hb concentration is a continuous variable. The relations of *EGLN1* haplotype frequencies to Hb concentration were examined using the negative binomial (NB) model via the R package “MASS” [49], with the conventional *p* value threshold.

We also tested the significance of differences in pairwise *D*′ or *r*^2^ values of focal SNPs between five 1KGP ethnic groups (American (AMR), East Asian (EAS), South Asian (SAS), European (EUR), and African (AFR)) using the Wilcoxon signed-rank test via the R package “coin” [50]. The statistical tests described above were performed using R software ver. 3.5.3 [51] in RStudio ver. 1.2.5019 [52].

### 2.6. Estimation of Haplotype Phase and Linkage Disequilibrium

We estimated the haplotype phase of the four SNPs around *EGLN1* in 99 Bolivian highlanders and linkage disequilibrium (LD) between pairs of SNPs using IMPUTE2 ver. 2.3.2 [53] and Haploview ver. 4.2, respectively. To generate phased haplotypes for the four SNPs around *EGLN1* in other populations, VCF data for 26 populations were retrieved from the 1KGP database using the Ensembl human genome database (genome assembly GRCh38.p13, http://www.ensembl.org/Homo_sapiens/Info/Index/ [54]). Haplotype bifurcation diagrams [55] in an ~200 kb genomic region around *EGLN1* were generated using the R package “rehh 3.0.1” [56, 57]. The datasets described above were converted into a suitable format for each program using R and Perl scripts.

*D*′ and *r*^2^ values for pairs of focal SNPs in the five 1KGP ethnic groups were examined using LDmatrix or LDpair in the LDlink web-based tool (https://ldlink.nci.nih.gov/ [58]). The allele frequency data of 26 populations in the 1KGP database were obtained from the Ensembl human genome database. To compare the allele frequencies of the four SNPs in highlander populations outside the Andes, we obtained the genotype data of Tibetan [15, 59] and Mongolian highlanders who had recently migrated to the Qinghai-Tibetan Plateau [60] from the Jorde Lab website (http://jorde.genetics.utah.edu/) under Published Data.

### 2.7. Assembly of Genomic Sequence around *EGLN1* in Bolivian Highlanders

We generated the binary alignment map (BAM) file for an ~436.3 kb genomic region around *EGLN1* in 42 Bolivian individuals using raw short-read data determined by Crawford et al. [24], which was deposited in the NCBI Sequence Read Archive (https://www.ncbi.nlm.nih.gov/sra/) under BioProject Accession Number PRJNA393593. Low-quality reads were filtered out using Trimmomatic ver. 0.39 [61] with the following parameters: LEADING:20, TRAILING:20, SLIDINGWINDOW:4:15, and MINLEN:75. After filtering, the Burrows-Wheeler Aligner ver. 0.7.17 software [62] was used to align the filtered paired-end reads to a human reference genome (GRCh37). The aligned BAM data were sorted and indexed using SAMtools ver. 1.9 [63], and then Picard tools (http://broadinstitute.github.io/picard/) were used to remove duplicate reads from the BAM output file. Finally, base quality scores were recalibrated using the Genome Analysis Toolkit [64].

### 2.8. Inferring Population Structure

To calculate allele frequencies from genotype likelihoods in the ~436.3 kb genomic region at 1q42.2, we used Analysis of Next Generation Sequencing Data (ANGSD) ver. 0.929 [65], which is a useful tool for minimizing potential genotype calling errors due to low-coverage sequencing data. Genotype likelihoods were calculated directly from the BAM files of the 42 Bolivian datasets described above and from each of the 50 individuals randomly sampled from the six 1KGP population datasets (PEL, CLM (Colombians from Medellin, Colombia), MXL (Mexican ancestry from Los Angeles USA), CEU (Utah residents with Northern and Western European ancestry), JPT (Japanese in Tokyo, Japan), and YRI (Yoruba in Ibadan, Nigeria)) retrieved from the 1KGP ftp site (ftp://ftp.1000genomes.ebi.ac.uk/vol1/ftp/phase3/data/).

NGSadmix [66] was applied to infer population structure from the calculated admixture proportions. Beagle format input data were generated from genotype likelihoods using the following parameters: -uniqueOnly 1, -remove_bads 1, -only_proper_pairs 1, -trim 0, -C 50, -baq 1, -minMapQ 30, -minQ 20, -minInd 10, -setMinDepth 60, -setMaxDepth -1, -doCounts 1, -GL 1, -doMajorMinor 4, -doMaf 1, -skipTriallelic 1, -doGlf 2, and -SNP_pval 1e-6. We then conducted NGSadmix analyses with a minimum minor allele frequency of 0.05 and assumed ancestral populations *K* of 3–5.

Using haplotype data generated from the VCF files of the 1KGP populations and the haplotypes estimated from the focal SNPs of the Bolivian cohort, we calculated *F*_ST_ values between pairs of three South AMR populations (Bolivia, PEL, and CLM) using Arlequin ver. 3.5.2.2 [67]. To further evaluate genetic differentiation between the six populations, including the three South AMRs, JPT, CEU, and YRI, we calculated three statistics (weighted *F*_ST_ [68], the population branch statistic (PBS) [18], and the modified PBS (PBS′) [24]) in the ~436.3 kb genomic region at 1q42.2 using estimated site frequency spectrums (SFSs). Firstly, we calculated site allele frequency likelihoods based on individual genotype likelihoods to estimate the SFS of each population or the joint SFS between populations in ANGSD. Secondly, we performed sliding window analyses of *F*_ST_ and PBS with 5 kb windows and 5 kb steps across the ~436.3 kb genomic region using the joint SFSs. Thirdly, we calculated PBS′ values from the PBS values [18], according to the formula proposed by Crawford et al. [24].

## 3. Results

### 3.1. Physiological Characteristics of Bolivian Highlanders

The study participants (49 men and 50 women) were enrolled from two cohorts located at altitudes of ~3700 m (the La Paz cohort) and ~4000 m (the El Alto cohort) in 2016. The characteristics of the study subjects in each cohort and the entire study population (entire cohort) are summarized in Table 1. The height, weight, and BMI of the 99 Bolivian highlanders were 159.5 ± 8.2cm, 62.2 ± 10.4kg, and 24.4 ± 3.6kg/m^2^, respectively. These anthropometric parameters differed significantly between the sexes, with the exception of BMI: mean height, 165.4 and 153.6 cm (*p* < 2.2 × 10^−16^, by Welch's *t*-test); mean weight, 68.1 and 56.3 kg (*p* = 5.9 × 10^−10^); and mean BMI, 24.9 and 23.9 kg/m^2^ (*p* = 0.1824) (in men and women, respectively).

**Figure 1:**
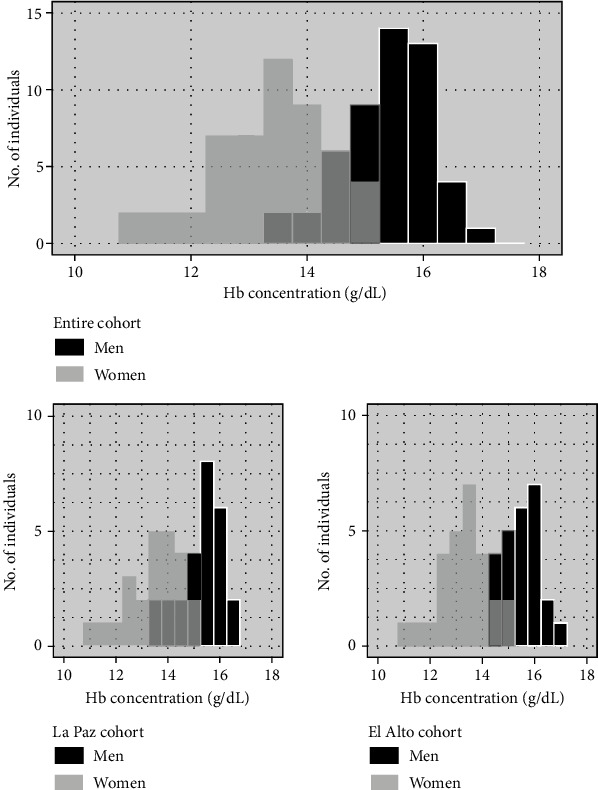
Count distribution of the estimated hemoglobin (Hb) concentration (g/dL) in men (black) and women (gray) in Bolivian cohorts.

The estimated SpO_2_ was significantly higher in the La Paz cohort than in the El Alto cohort (*p* = 0.0027). Conversely, SBP and DBP were significantly higher in the El Alto cohort than in the La Paz cohort (*p* = 1.8 × 10^−9^‐0.0020). While current smoking rates were 2% in each cohort, the percentage of individuals who drank alcohol was higher in the La Paz cohort than in the El Alto cohort (27.1 vs. 7.8%; *p* = 0.0156, by Fisher's exact test). The estimated Hb concentration in men was significantly higher than that in women in the two cohorts (*p* = 9.5 × 10^−13^‐5.0 × 10^−9^, by Welch's *t*-test). The Hb concentration in men was higher in the El Alto cohort than in the La Paz cohort, whereas Hb levels in women were similar in both cohorts. No significant differences in the Hb levels were observed between participants of the same sex residing at the different altitudes (*p* = 0.27‐0.86). The distributions of Hb levels in men and women are shown in Figure 1.

### 3.2. Genotyping of Four SNPs around *EGLN1* in Bolivian Highlanders

In this study, we genotyped five SNPs (rs2486740, rs508618, rs12097901, rs186996510, and rs1769792) around *EGLN1* in 99 individuals from La Paz and El Alto in Bolivia, although rs186996510 was removed for further analyses due to the absence of genetic variation. The remaining four SNPs were selected as tag SNPs covering a genomic region of ~81 kb around *EGLN1* (Supplement Figure 1). None of the SNPs showed significant deviation from HWE (*p* = 0.15‐1.00; Table 2). The mean observed and expected heterozygosities of the four SNPs in the entire cohort were 0.415 and 0.408, respectively.

The SNP rs12097901, which is located in exon 1 of *EGLN1*, is involved in an amino acid change (C127S), whereas the other SNPs are located in intronic (rs2486740 and rs508618) and intergenic (rs1769792) regions. According to the CADD database [48], nucleotide substitution at each SNP appears to have a weak effect on protein function (CADDscaledC − scores = 0.19–3.47), with the exception of rs12097901 (CADDscaledC − score = 14.59). We also surveyed the allele frequencies of the four SNPs across 26 populations from the 1KGP via the Ensembl human genome database (Supplement Table 2), finding that the frequency of the major allele (G) at rs1769792 was remarkably higher in the Bolivian cohort (60.6%) than in Asian (0.5–16.1%) and European populations (18.7–29.0%). This suggests that the allele frequency increased after the Native American population split from other populations.

Next, we examined LD between the four SNPs in 99 Bolivian highlanders (Supplement Figure 1). There was moderate LD between three SNP pairs (rs2486740 vs. rs508618, rs2486740 vs. rs1769792, and rs12097901 vs. rs1769792; *D*′ = 0.77‐1.00, *r*^2^ = 0.32‐0.56). We also examined LD between the corresponding pairs of SNPs in five ethnic groups (AMR, EAS, SAS, EUR, and AFR), using 1KGP datasets (Supplement Table 3) in LDmatrix, an LDlink web-based application [58]. *D*′ and *r*^2^ values did not differ significantly between the ethnic groups (*p* = 0.094‐1.000, by Wilcoxon's signed-rank test).

### 3.3. Association between Physiological Parameters and Four SNPs around *EGLN1*

In this study, we investigated the association between the four SNPs around *EGLN1* and the physiological parameters of 99 Bolivian highlanders by the linear regression test and Welch's *t*-test using the additive and dominant genetic models, respectively (Supplement Table 4). In the La Paz cohort, rs508618 and rs1769792 were significantly (*p* = 0.008‐0.009) associated with Hb levels in the additive and dominant genetic models, respectively, while rs1769792 was related to Hb levels in the entire cohort in a borderline significant manner (*p* = 0.015). In this study, we found no significant association between Hb concentration and rs12097901, which was previously associated with Hb levels in Tibetans [29]. The relations of rs1769792 to SpO_2_ (*p* = 0.016) and rs12097901 to heart rate (*p* = 0.015) were borderline significant in the La Paz and El Alto cohorts, respectively. There was no statistically significant association with the other physiological parameters.

To confirm the association between the two candidate SNPs, rs508618 and rs1769792, and Hb levels in the entire cohort, we used the GLM after adjusting for the potential confounders, age, sex, and altitude. These analyses revealed that Hb levels were not associated with the rs508618 genotype in additive or dominant genetic models (*p* = 0.365‐0.512); however, rs1769792 genotype frequency was related to Hb concentration in the dominant genetic model (*p* = 0.040), although the relation did not reach the significance level in the additive model (*p* = 0.148). In both the La Paz and El Alto cohorts, Hb levels were higher in individuals with the GG genotype (mean Hb, 14.8–15.1 g/dL) at rs1769792 than in those with GA or AA genotypes (mean Hb, 14.0–14.7 g/dL; Supplement Figure 2 and Supplement Table 5). Moreover, mean Hb concentrations were higher in individuals with major alleles at all SNPs examined than in those with the corresponding minor alleles (Table 3).

According to the GTEx Portal and The Human Protein Atlas databases, *EGLN1* is predominantly expressed in skeletal muscle; therefore, we investigated the relationship between gene expression in this tissue and the focal SNPs in the GTEx database. The eQTL analysis from the database revealed that the Andean-dominant allele (G) of rs1769792 and the T allele of rs2486740 significantly reduced *EGLN1* expression levels in skeletal muscle (rs1769792, *p* = 1.5 × 10^−5^; rs2486740, *p* = 1.2 × 10^−9^). However, there was no significant association between *EGLN1* expression in this tissue and rs508618 or rs12097901.

### 3.4. Combined Effects of *EGLN1* SNPs on Hb Levels

**Figure 2:**
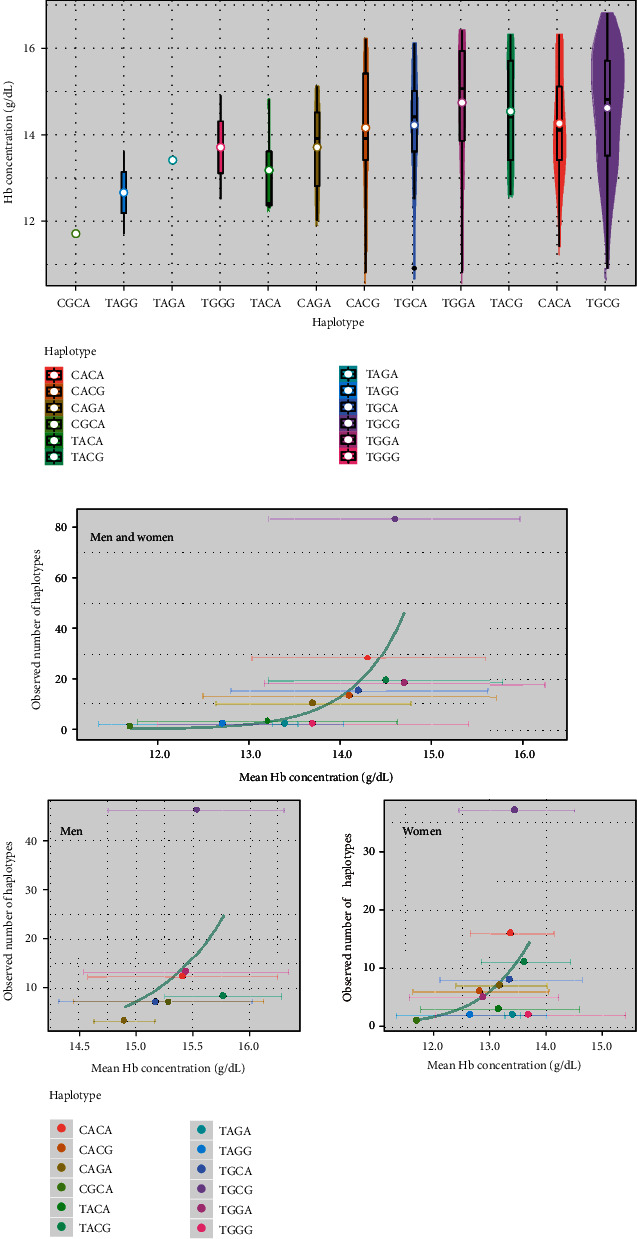
(a) Hemoglobin (Hb) concentrations (g/dL) in individuals with different haplotypes estimated from four *EGLN1* SNPs (rs2486740, rs508618, rs12097901, and rs1769792) in the entire cohort. White dots represent mean Hb concentration values and bold black bars represent median values. The Hb measurement data were obtained by using a noninvasive method. (b) Relationship between the estimated Hb concentration and *EGLN1* haplotype frequency in all subjects (upper graph) and male or female subjects (bottom graphs) in the entire cohort. Hb levels are represented as the mean ± standarddeviation of the mean. The bold blue line represents the regression line.

To examine the combined effects of the four *EGLN1* SNPs on Hb levels, we estimated their phased haplotypes. Mean Hb levels of individuals with each haplotype are shown in Figure 2(a) and Table 4. In the entire cohort, *EGLN1* haplotype frequencies increased with the mean Hb concentration (Figure 2(b)), and the NB model showed that the association was statistically significant (*p* = 2.0 × 10^−7^). This association was replicated even though the haplotype frequency and Hb data were separated by sex (*p* = 0.012‐0.016) or cohort (*p* = 4.2 × 10^−4^‐0.020). The relationships between the haplotype frequencies and Hb levels divided by sex and cohort are shown in Figure 2(b) and Supplement Figure 3, respectively.

**Figure 3:**
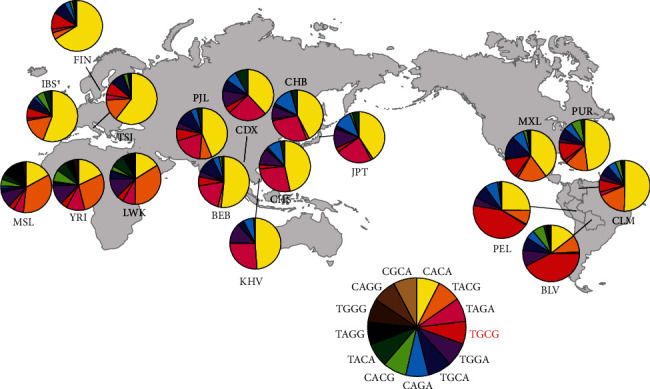
Frequency distributions of *EGLN1* haplotypes (rs2486740, rs508618, rs12097901, and rs1769792). BLV: Bolivian from La Paz and El Alto, Bolivia; CLM: Colombians from Medellin, Colombia; MXL: Mexican ancestry from Los Angeles, USA; PEL: Peruvians from Lima, Peru; PUR: Puerto Ricans from Puerto Rico; CDX: Chinese Dai in Xishuangbanna, China; CHB: Han Chinese in Beijing, China; CHS: Southern Han Chinese; JPT: Japanese in Tokyo, Japan; KHV: Kinh in Ho Chi Minh City, Vietnam; BEB: Bengali from Bangladesh; PJL: Punjabi from Lahore, Pakistan; FIN: Finnish in Finland; IBS: Iberian population in Spain; TSI: Toscani in Italia; LWK: Luhya in Webuye, Kenya; MSL: Mende in Sierra Leone; YRI: Yoruba in Ibadan, Nigeria. Haplotype frequencies, with the exception of BLV, were estimated using the variant call format (VCF) data obtained from the 1000 Genomes Project. The “TGCG” haplotype shown in red is predominantly distributed in Native American highlanders.

The “TGCG” (rs2486740, rs508618, rs12097901, and rs1769792 alleles) haplotype had the highest frequency (42.4%) in the Bolivian cohort and consisted of all major alleles with higher Hb levels than the corresponding minor allele for each SNP (Table 4). Moreover, the mean Hb concentration of individuals carrying the dominant haplotype was the second highest in the entire cohort. Irrespective of sex, this haplotype dominated (37.0–47.9%), with the second and third highest Hb levels in men and women, respectively (Table 5). Next, we assessed frequencies of the corresponding *EGLN1* haplotypes using the VCF data of 26 populations in the 1KGP database and compared them to the Bolivian dataset from this study. Intriguingly, the “TGCG” haplotype was predominantly distributed in the Andean Altiplano (42.4–43.5%), with a low haplotype frequency in other populations (0.5–13.9%) including CLM (7.4%; Figure 3 and Supplement Table 6).

In a previous genome-wide selection scan in Andeans, rs1769792 showed the strongest signal of positive selection in the *EGLN1* genomic region [26]. To estimate the decay of LD from the core haplotype carrying the Andean-dominant allele (G) of rs1769792, we generated haplotype bifurcation plots [55] in an ~200 kb genomic region containing *EGLN1* for four populations (PEL, CLM, CEU, and JPT) from the 1KGP database (Supplement Figure 4). The bifurcation plots revealed that the G allele haplotype in PEL had the longest-range LD among the four populations, although the LD appeared to have broken down somewhat. The bifurcation plot for JPT showed a clear breakdown of haplotype homozygosity for the G allele. Haplotype bifurcation patterns were relatively similar between CLM and CEU, and this may reflect admixture events between Europeans and Native Americans [69, 70].

### 3.5. Inference of Population Structure Using *EGLN1* Genetic Data

Using the *EGLN1* haplotype frequency data based on the focal SNPs, we calculated the *F*_ST_ [71], an index of genetic differentiation, between three South AMR populations: the Bolivian cohort, PEL, and CLM. The analysis indicated a low level of genetic differentiation between the Bolivian and PEL populations (*F*_ST_ = 0.003, *p* = 0.225) but statistically significant levels of genetic differentiation between the CLM and Bolivian or PEL populations (*F*_ST_ = 0.134‐0.187, *p* < 1.0 × 10^−5^). There was no significant genetic differentiation between the La Paz and El Alto cohorts (*F*_ST_ = 0.004, *p* = 0.288). According to the result of the *F*_ST_-based analysis, these cohorts are likely to be genetically homogenous.

Next, we inferred the genetic structure of seven human populations (50 randomly sampled individuals in each of six 1KGP populations (PEL, CLM, MXL, CEU, JPT, and YRI) and 42 Bolivians [24]) using the NGSadmix program, based on genotype likelihoods in a 436.3 kb genomic region around *EGLN1*. The NGSadmix analysis showed that the genetic structure of two lowland AMR populations (CLM and MXL), estimated from admixture proportions, was more similar to CEU with European ancestry than highland Native American (HNA; Bolivian and PEL) populations (Supplement Figure 5). In addition, genetic components of YRI with African ancestry appeared to be included in the lowland AMR populations. These findings suggest that admixture events between lowland AMR natives and Europeans or Africans [69, 70] have affected the genomic region around *EGLN1*, but had lesser impact on the genomic region in HNA.

**Figure 4:**
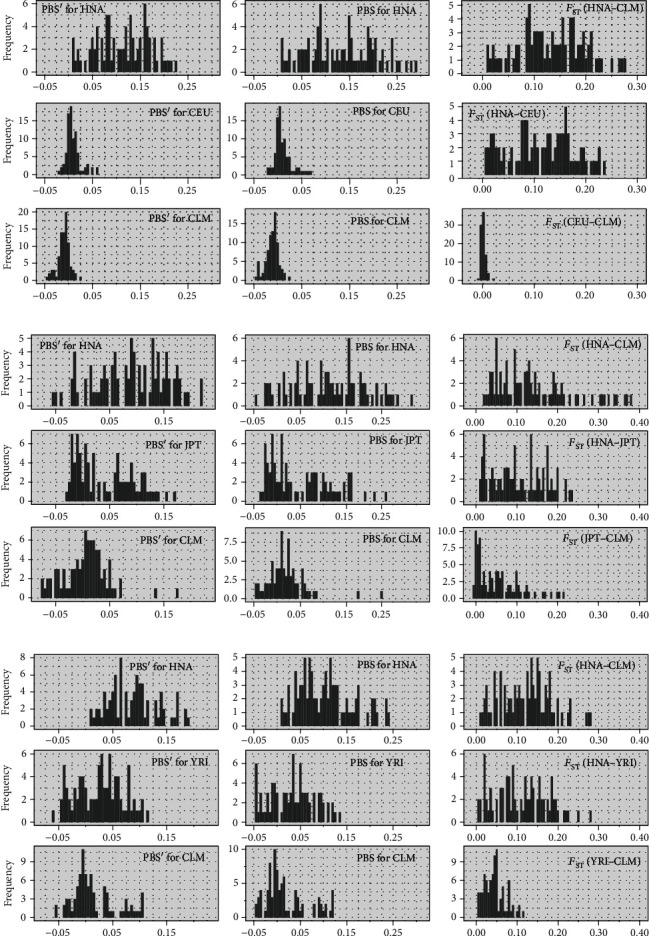
Frequency distributions of three statistical values (weighted *F*_ST_, population branch statistic (PBS), and the modified PBS (PBS′)) in an ~436.3 kb genomic region containing *EGLN1*, calculated using the estimated allele frequency data of three population triplets: (a) HNA vs. CLM vs. CEU; (b) HNA vs. CLM vs. JPT; (c) HNA vs. CLM vs. YRI. HNA: highland Native Americans; CLM: Colombians from Medellin, Colombia; CEU: Utah residents with Northern and Western European Ancestry; JPT: Japanese in Tokyo, Japan; YRI: Yoruba in Ibadan, Nigeria.

To investigate the Andean population structure, we performed sliding window analyses (5 kb windows and 5 kb steps) on three statistics, weighted *F*_ST_ [68], PBS [18], and PBS′ [24], throughout the ~436.3 kb genomic region using the estimated allele frequency data in three population triplets (HNA-CLM-CEU, HNA-CLM-JPT, and HNA-CLM-YRI; Supplement Tables 7–9). Although PBS-based statistics are normally used to detect signals of positive natural selection, we used them to compare genetic relatedness between HNA and other lowland populations. Comparisons of the three statistics between populations showed high PBS and PBS′ values for HNA and high *F*_ST_ values between HNA and lowlander populations (Figures 4(a)–4(c)). Here, “PBS(′) value for HNA” denotes that the allele frequency at the site is assumed to have changed substantially in the HNA population. Although Colombia is geographically close to Bolivia and Peru, our analyses showed that the CLM population appeared to be more closely related to CEU or YRI than HNA within the *EGLN1* genomic region because of similar PBS or PBS′ frequency distributions and low *F*_ST_ values between these populations (Figures 4(a) and 4(c)), suggesting admixture events between lowland AMR natives and Europeans or Africans.

**Figure 5:**
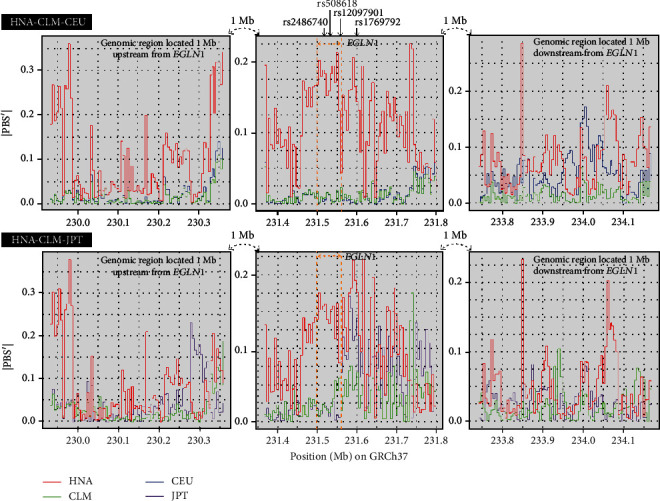
Modified population branch statistic (PBS′) proposed by Crawford et al. [24] in an ~436.3 kb genomic region containing *EGLN1* or 436.3 kb genomic regions located 1 Mb upstream or downstream of *EGLN1*, calculated using the estimated allele frequency data for two population triplets (HNA, CLM, and CEU or JPT). |PBS′| values are shown for a sliding window with 5 kb windows and 5 kb steps. Red, green, blue, and purple lines represent PBS′ values in HNA, CLM, CEU, and JPT populations, respectively. The positions of the four SNPs examined are indicated with an arrow. The horizontal axis represents chromosomal position (NCBI build GRCh37). HNA: highland Native Americans; CLM: Colombians from Medellin, Colombia; CEU: Utah residents with Northern and Western European Ancestry; JPT: Japanese in Tokyo, Japan.

The sliding window analyses of PBS′ values for two of the population triplets (HNA-CLM-CEU and HNA-CLM-JPT) in the ~436.3 kb genomic region are shown in Figure 5. PBS′ values for HNA (PBS′ = 0.114 ± 0.055) were consistently higher across this genomic region than in the CLM (PBS′ = −0.010 ± 0.013) and CEU (PBS′ = 0.010 ± 0.015) populations. Moreover, the PBS′ analysis based on the HNA-CLM-JPT dataset showed that PBS′ values in an ~94.2 kb genomic segment nearby *EGLN1* were considerably higher in the HNA population (PBS′ = 0.137 ± 0.023) than in the CLM (PBS′ = 0.025 ± 0.015) and JPT (PBS′ = −0.014 ± 0.011) populations. In genomic regions located 1 Mb upstream or downstream of *EGLN1*, PBS′ values for HNA were lower than those around *EGLN1*, although the PBS′ values of several fragments were high (Figure 5 and Supplement Tables 10–11). These results suggest that the population structure in Andeans based on genetic markers around *EGLN1* differs largely from that in the other populations.

## 4. Discussion

It has previously been reported that Andean highlanders display a higher Hb concentration than Ethiopian and Tibetan highlanders [11, 12, 72]. Although recent genome-wide scans have detected signals of positive natural selection in several genomic regions in Andean highlanders [22–24], no studies have yet reported candidate genes responsible for the elevated Hb levels in Andeans. In this study, we found that estimated *EGLN1* haplotype frequencies were significantly correlated with Hb concentration. These findings are the first report showing significant relationships between genetic polymorphisms and the elevated Hb levels in Andeans.

Previous studies have reported the mean Hb levels of 17.5–19.2 and 14.5–17.8 g/dL for Bolivian men and women living at altitudes of 3600–4000 m [12, 39, 73], respectively. In our study, the mean Hb levels were 15.4 and 13.3 g/dL in men and women, respectively. Since the Hb measurement data are estimated values (see Materials and Methods), the measured values were likely underestimated. However, it is unlikely to critically affect the comparison of Hb levels among the study subjects because the Hb levels for all subjects were measured in the same manner. This study is aimed at finding the association between *EGLN1* genetic variants and Hb levels in Bolivian highlanders; thus, the relative values of the Hb measurement can be sufficient to detect the association in the Bolivian cohorts examined.

Previous studies have reported that *EGLN1* is associated with Hb concentration in Tibetan highlanders [15, 19, 29] but has no relationship with Hb levels in Andean highlanders [39]. Henrich et al. [74] found that possible Tibetan-adaptive alleles were absent at *EGLN1* rs186996510 or in low frequency at rs12097901 (12.7%) in Andean highlanders. Our study also found similar allele frequencies of the two SNPs in highland and lowland AMR natives as well as those reported in Peruvian Quechua highlanders [25]. In addition, the major allele (G) of rs1769792 in the Bolivian cohort was minor (0.016–0.117) in Tibetan [15, 59] and Deedu Mongolian [60] highlander populations. Therefore, genetic mechanisms of high-altitude adaptation may be different between Tibetan and Andean highlander populations.

In Peruvian Quechua highlanders, individuals with the rs1769793-T allele showed significantly higher VO_2_max values than those with its counterpart allele [25]. According to LDpair in LDlink, the rs1769793-T allele was in significant LD (*D*′ = 1.00, *r*^2^ = 0.90, *p* < 0.0001) with the rs1769792-G allele in PEL, suggesting that the VO_2_max and Hb levels of individuals with these alleles are high, although it remains unclear which SNPs actually affect the pulmonary and hematological functions in Andeans. Further analysis is required to clarify the functional relevance of *EGLN1* SNPs to Hb concentration. According to the GTEx Portal database, *EGLN1* expression levels were low in skeletal muscle with the rs1769792-G allele, compared to the counterpart “A” allele. The downregulation of the PHD2 protein encoded by *EGLN1* enhances HIF-*α* activity, resulting in increased Hb levels via erythropoiesis. Thus, the negative correlation between *EGLN1* expression and Hb levels in individuals with the high-altitude allele is reasonable. In fact, individuals with the rs1769792-G allele in the Bolivian cohort displayed a higher Hb concentration than those with the counterpart allele.

Bigham et al. [26] independently detected signals of positive selection in the genomic regions around *EGLN1* in Andean and Tibetan populations, with rs1769792 having the highest ranked signal in this genomic region in the Andeans. This SNP is located between rs2790859 and rs961154 that have shown high integrated haplotype scores [4] in Tibetan highlanders [15]. The three SNPs are located within ~2.8 kb of an intergenic region near *EGLN1*; therefore, one may hypothesize that the genomic segment around rs1769792 is involved in regulating gene expression and may be responsible for high-altitude adaptation. In this study, rs1769792-G allele frequencies were higher in the Bolivian and PEL populations than in three other AMR lowlander populations (CLM, MXL, and PUR); however, the difference in allele frequencies was unremarkable. In addition, the haplotype bifurcation analysis revealed that haplotypes carrying the rs1769792-G allele in the PEL population had the longest LD range, but some breakdown of haplotype homozygosity was observed. Given that natural selection has acted on the *EGLN1* genomic region in Andean highlanders [26], selection may still be ongoing because human populations colonized the Andean Altiplano thousands of years ago [22, 75], which is relatively recent in evolutionary terms. Alternatively, the target SNPs of *EGLN1* may contribute toward polygenic adaptation from standing genetic variation in response to the hypobaric hypoxia environment in Andean highlanders, since polygenic adaptation plays an important role in high-altitude adaptation in modern humans [76]. According to CADD scores, the effects of most *EGLN1* SNPs examined on PHD2 protein function appear to be small. Therefore, the combined effects of each SNP may play an important role in Hb concentrations in Andean highlanders because of polygenic inheritance or epistasis.

In the Bolivian cohort, the *EGLN1* haplotype frequencies were significantly correlated with Hb levels, irrespective of sex or altitude, suggesting that the relatively high Hb levels in Andean highlanders can be partly characterized by *EGLN1* genetic variants. In addition, the “TGCG” haplotype consisting of all major alleles was predominantly distributed in the Andean Altiplano and appeared to have expanded rapidly in native Andean highlanders. The Andean-dominant haplotype was also observed at low frequency levels in other modern human populations; therefore, the origin of the “TGCG” haplotype could be traced back to the common ancestor of modern humans.

We also found that the *EGLN1* genomic region appeared to be less influenced by European or African admixture in Andean highlanders (i.e., HNA) than in lowland Native Americans, consistent with the results of admixture analyses using whole genome sequence data [24]. It is possible that Andean highlander populations have a higher proportion of Native American ancestry than the lowlander populations because individuals with European or African ancestry were less likely to migrate (individuals with African ancestry may have been brought) into highland populations due to the severe high-altitude conditions. In addition, sliding window analyses of PBS′ suggested that the population structure based on *EGLN1* genetic markers in Andean highlanders differs largely from that of other human populations including the closely related population. In fact, the TGCG haplotype frequency was markedly higher in Bolivia and PEL than CLM. It is possible that haplotypes carrying the TGCG-motif with high Hb levels contribute to the genetic components in Andeans. Therefore, the Andean-specific genetic components around *EGLN1* may affect physiological phenotypes of Andean highlanders in response to hypobaric hypoxia.

There were several limitations to this study. Firstly, the sample size is not sufficient to detect the association between SNPs and phenotypes with a high statistical power. Secondly, the four tag SNPs examined in this study are in LD with neighboring genetic variants across *EGLN1*; therefore, it is possible that these other genetic variants may actually affect Hb dynamics. Thirdly, since Hb concentrations were measured using a noninvasive method, they are estimated values. Lastly, given that polygenic adaptation is important for high-altitude adaptation and that phenotypic plasticity in response to hypobaric hypoxia is likely driven by epigenetics, genome- and epigenome-wide association studies would improve our understanding of hypobaric hypoxia adaptability in present-day Andeans.

## 5. Conclusion

In a Bolivian cohort of 99 healthy men and women, we found that the estimated *EGLN1* haplotype frequencies were significantly correlated with Hb levels in the Bolivians. Our study is the first to show a significant relationship between genetic variants and Hb levels in Andean highlanders. We also found that the long-range haplotypes with the “TGCG” motif at the focal SNPs may have rapidly expanded in this highlander population because these haplotypes may confer adaptive phenotypes against hypobaric hypoxia stress. In conclusion, the combined effects of *EGLN1* SNPs could be responsible for the intrinsic Hb dynamics of Andean highlanders, in concert with the effects of other genetic variants related to physiological responses to hypobaric hypoxia.

## Figures and Tables

**Table 1:** Characteristics of the study subjects in the Bolivian cohorts.

Characteristic	Entire^a^	La Paz	El Alto	*p* value^b^
No. of subjects	99	48	51	
Sex (no. of men/women)	49/50	24/24	25/26	1.0000
Age (years)^c^	25.1 ± 3.2	24.9 ± 3.0	25.3 ± 3.5	0.4390
Height (cm)^c^	159.5 ± 8.2	161.2 ± 8.2	157.9 ± 8.0	0.0281^∗^
Weight (kg)^c^	62.2 ± 10.4	63.6 ± 11.1	61.0 ± 9.7	0.1034
BMI (kg/m^2^)^c^	24.4 ± 3.6	24.3 ± 3.2	24.5 ± 3.9	0.9073
Hb concentration (g/dL)^c,d^	14.4 ± 1.4	14.3 ± 1.4	14.4 ± 1.4	0.9703
Hb in men (g/dL)^c,d^	15.4 ± 0.8	15.3 ± 0.8	15.6 ± 0.7	0.2673
Hb in women (g/dL)^c,d^	13.3 ± 1.0	13.4 ± 1.0	13.3 ± 1.0	0.8566
SpO_2_ (%)^c,d^	90.5 ± 2.6	91.3 ± 2.4	89.7 ± 2.5	0.0027^∗∗^
Heart rate (bpm)^c^	77.1 ± 11.9	79.3 ± 13.2	75.0 ± 10.2	0.0740
SBP (mmHg)^c^	113.7 ± 12.2	109.8 ± 11.6	117.3 ± 11.7	0.0020^∗∗^
DBP (mmHg)^c^	65.8 ± 8.6	60.9 ± 6.6	70.5 ± 7.7	1.8 × 10^−9∗∗^
Current smoking (%)	2.0	2.1	2.0	1.0000
Alcohol drinking (%)	17.2	27.1	7.8	0.0156^∗^
Exercise (%)	47.5	50.0	45.1	0.6894

Abbreviations: BMI—body mass index; Hb—hemoglobin; SpO_2_—percutaneous arterial oxygen saturation; SBP—systolic blood pressure; DBP—diastolic blood pressure. ^a^Combined cohort of La Paz and El Alto cohorts. ^b^Welch's *t*-test and Fisher's exact test were used to test differences in quantitative and categorical data, respectively, between the La Paz and El Alto cohorts. Based on Bonferroni's correction, a *p* value of < 0.0038 (0.05/13) was considered statistically significant. ^c^Quantitative data are presented as the mean ± standarddeviation of the mean. ^d^Estimated value obtained by using a noninvasive method. ^∗^*p* < 0.05. ^∗∗^*p* < 0.01.

**Table 2:** Summary of the four *EGLN1* SNPs examined in this study.

RefSNP ID	Position	Cohort	*N* ^d^	Allele frequency^e^		*H*_obs_	*H*_exp_	HWE (*p* value)^f^
				Major	Minor			
rs2486740	1 : 231517552^a^	Entire^c^	99	T: 0.73 (145)	C: 0.27 (53)	0.37	0.39	0.61
	1 : 231381806^b^	La Paz	48	T: 0.71 (68)	C: 0.29 (28)	0.38	0.41	0.50
		El Alto	51	T: 0.75 (77)	C: 0.25 (25)	0.37	0.37	1.00
rs508618	1 : 231532312^a^	Entire^c^	99	G: 0.61 (120)	A: 0.39 (78)	0.46	0.48	0.83
	1 : 231396566^b^	La Paz	48	G: 0.55 (53)	A: 0.45 (43)	0.52	0.49	1.00
		El Alto	51	G: 0.66 (67)	A: 0.34 (35)	0.41	0.45	0.54
rs12097901	1 : 231557255^a^	Entire^c^	99	C: 0.83 (164)	G: 0.17 (34)	0.55	0.48	0.21
	1 : 231421509^b^	La Paz	48	C: 0.81 (78)	G: 0.19 (18)	0.60	0.49	0.15
		El Alto	51	C: 0.84 (86)	G: 0.16 (16)	0.49	0.46	0.77
rs1769792	1 : 231598618^a^	Entire^c^	99	G: 0.61 (120)	A: 0.39 (78)	0.28	0.28	1.00
	1 : 231462872^b^	La Paz	48	G: 0.57 (55)	A: 0.43 (41)	0.33	0.30	1.00
		El Alto	51	G: 0.64 (65)	A: 0.36 (37)	0.24	0.26	0.59

Abbreviations: SNP—single-nucleotide polymorphism; *H*_obs_—observed heterozygosity; *H*_exp_—expected heterozygosity; HWE—Hardy-Weinberg equilibrium. ^a^Chromosomal position in NCBI build GRCh37. ^b^Chromosomal position in NCBI build GRCh38. ^c^Combined cohort of La Paz and El Alto cohorts. ^d^The number of individuals. ^e^Values indicate allele frequencies, with the observed numbers indicated in parentheses. ^f^Probability of a genetic variant whose genotype distribution does not deviate from HWE.

**Table 3:** Hemoglobin levels of Bolivian highlanders with different genotypes of four SNPs around *EGLN1.*

RefSNP ID	Position	Genotype	*N* ^c^	Hb level (g/dL)^d^
rs2486740	1 : 231517552^a^	TT	54	14.6 ± 1.4
	1 : 231396566^b^	TC	36^e^	14.2 ± 1.4^e^
		CC	8	13.8 ± 1.3

rs508618	1 : 231532312^a^	GG	37	14.7 ± 1.4
	1 : 231396566^b^	GA	45^e^	14.3 ± 1.4^e^
		AA	16	13.8 ± 1.3

rs12097901	1 : 231557255^a^	CC	67^e^	14.5 ± 1.4^e^
	1 : 231421509^b^	CG	28	14.2 ± 1.6
		GG	3	13.8 ± 0.9

rs1769792	1 : 231598618^a^	GG	33	14.8 ± 1.2
	1 : 231462872^b^	GA	53^e^	14.1 ± 1.5^e^
		AA	12	14.4 ± 1.0

Abbreviations: SNP—single-nucleotide polymorphism. Hb—hemoglobin. ^a^Chromosomal position in NCBI build GRCh37. ^b^Chromosomal position in NCBI build GRCh38. ^c^The number of subjects. ^d^Estimated values obtained by using a noninvasive method (the mean ± standarddeviation of the mean). ^e^One individual was removed due to a lack of Hb measurement.

**Table 4:** Haplotype frequencies estimated from four SNPs around *EGLN1* and hemoglobin levels in Bolivian highlanders.

Haplotype^a^	2*N*^b^	Frequency	Hb level (g/dL)^c^
TGCG	83^d^	0.424	14.60 ± 1.37^d^
CACA	28^d^	0.146	14.25 ± 1.28^d^
TACG	19	0.096	14.53 ± 1.28
TGGA	18	0.091	14.73 ± 1.54
TGCA	15	0.076	14.21 ± 1.41
CACG	13	0.066	14.15 ± 1.61
CAGA	10	0.051	13.70 ± 1.07
TACA	3	0.015	13.17 ± 1.42
TGGG	2	0.010	13.70 ± 1.70
TAGA	2	0.010	13.40 ± 0.14
TAGG	2	0.010	12.65 ± 1.34
CGCA	1	0.005	11.70 ± 0.00

Abbreviations: SNP—single-nucleotide polymorphism. Hb—hemoglobin. ^a^Haplotype consists of four SNPs examined (from left, rs2486740, rs508618, rs12097901, and rs1769792). ^b^The number of chromosomes. ^c^Estimated values obtained by using a noninvasive method (the mean ± standarddeviation of the mean). ^d^One individual was removed due to a lack of Hb measurement.

**Table 5:** Haplotype frequencies estimated from four SNPs around *EGLN1* and Hb levels in 48 male and 50 female Bolivian highlanders.

Sex	Haplotype^a^	2*N*^b^	Frequency	Hb level (g/dL)^c^
Men	TGCG	46^d^	0.479	15.53 ± 0.77^d^
	TGGA	13	0.135	15.41 ± 0.83
	CACA	12^d^	0.125	15.76 ± 0.51^d^
	TACG	8	0.083	15.44 ± 0.89
	TGCA	7	0.073	15.17 ± 0.84
	CACG	7	0.073	15.29 ± 0.83
	CAGA	3	0.031	14.90 ± 0.26

Women	TGCG	37	0.370	13.46 ± 1.04
	CACA	16	0.160	13.38 ± 0.75
	TACG	11	0.110	13.63 ± 0.80
	TGCA	8	0.080	12.88 ± 1.34
	CAGA	7	0.070	13.36 ± 1.27
	CACG	6	0.060	12.82 ± 1.22
	TGGA	5	0.050	13.19 ± 0.82
	TACA	3	0.030	13.17 ± 1.42
	TGGG	2	0.020	13.70 ± 1.70
	TAGA	2	0.020	13.40 ± 0.14
	TAGG	2	0.020	12.65 ± 1.34
	CGCA	1	0.010	11.70 ± 0.00

Abbreviations: SNP—single-nucleotide polymorphism. Hb—hemoglobin. ^a^Haplotype consists of four SNPs examined (from left, rs2486740, rs508618, rs12097901, and rs1769792). ^b^The number of chromosomes. ^c^Estimated values obtained by using a noninvasive method (the mean ± standarddeviation of the mean). ^d^One individual was removed due to a lack of Hb measurement.

## Data Availability

All data generated or analyzed during this study are included in this published article and its supplementary information files.

## Supplementary Materials

Supplementary 1 Supplement Table 1: genotype data for *EGLN1* SNPs in each individual. Supplement Table 2: allele frequencies of four SNPs (rs2486740, rs508618, rs12097901, and rs1769792) around *EGLN1* in different human populations. Supplement Table 3: linkage disequilibrium (*r*^2^ below the diagonal and *D*′ above the diagonal) of four SNPs around *EGLN1* in five ethnic groups from the 1000 Genomes Project. Supplement Table 4: statistical tests for the association between *EGLN1* genotypes and hematological traits. Supplement Table 5: hematological characteristics of 99 Bolivian highlanders with different genotypes of four SNPs around *EGLN1*. Supplement Table 6: observed number of haplotypes for four SNPs (rs2486740, rs508618, rs12097901, and rs1769792) around *EGLN1* in different human populations. Supplement Table 7: three statistical values (weighted *F*_ST_, PBS, and PBS′) in an ~436.3 kb genomic region containing *EGLN1* (a sliding window with 5 kb windows and 5 kb steps), calculated using the estimated allele frequency data of three populations (HNA, CLM, and CEU). Supplement Table 8: three statistical values (weighted *F*_ST_, PBS, and PBS′) in an ~436.3 kb genomic region containing *EGLN1* (a sliding window with 5 kb windows and 5 kb steps), calculated using the estimated allele frequency data of three populations (HNA, CLM, and JPT). Supplement Table 9: three statistical values (weighted *F*_ST_, PBS, and PBS′) in an ~436.3 kb genomic region containing *EGLN1* (a sliding window with 5 kb windows and 5 kb steps), calculated using the estimated allele frequency data of three populations (HNA, CLM, and YRI). Supplement Table 10: PBS′ in an ~436.3 kb genomic region located 1 Mb upstream of *EGLN1* (a sliding window with 5 kb windows and 5 kb steps), calculated using the estimated allele frequency data for two population triplets (HNA, CLM, and JPT or CEU). Supplement Table 11: PBS′ in an ~436.3 kb genomic region located 1 Mb downstream of *EGLN1* (a sliding window with 5 kb windows and 5 kb steps), calculated using the estimated allele frequency data for two population triplets (HNA, CLM, and JPT or CEU).

Supplementary 2 Supplement Figure S1: the physical position and linkage disequilibrium of four SNPs across an ~81 kb genomic region around *EGLN1* in 99 Bolivian highlanders. Supplement Figure S2: the estimated hemoglobin (Hb) concentrations (g/dL) in individuals with different *EGLN1* rs1769792 genotypes in La Paz, El Alto, and the entire cohort. Supplement Figure S3: relationship between the estimated hemoglobin (Hb) concentration (g/dL) and *EGLN1* haplotype frequency in the Bolivian cohorts. Supplement Figure S4: haplotype bifurcation plots for an ~200 kb genomic region containing *EGLN1*. Supplement Figure S5: genetic structure analysis of 342 individuals from BLV, PEL, CLM, MXL, CEU, JPT, and YRI populations.
