# Supplementary material for: Effect of EGLN1 Genetic Polymorphisms on Hemoglobin Concentration in Andean Highlanders
Source: Biomed Res Int. 2020 Nov 15;2020:3436581. doi: 10.1155/2020/3436581 (PMC7686849; doi:10.1155/2020/3436581)
Supplement: Supplementary 1 — Supplement Table 1: genotype data for EGLN1 SNPs in each individual. Supplement Table 2: allele frequencies of four SNPs (rs2486740, rs508618, rs12097901, and rs1769792) around EGLN1 in different human populations. Supplement Table 3: linkage disequilibrium (r2 below the diagonal and D′ above the diagonal) of four SNPs around EGLN1 in five ethnic groups from the 1000 Genomes Project. Supplement Table 4: statistical tests for the association between EGLN1 genotypes and hematological traits. Supplement Table 5: hematological characteristics of 99 Bolivian highlanders with different genotypes of four SNPs around EGLN1. Supplement Table 6: observed number of haplotypes for four SNPs (rs2486740, rs508618, rs12097901, and rs1769792) around EGLN1 in different human populations. Supplement Table 7: three statistical values (weighted FST, PBS, and PBS′) in an ~436.3 kb genomic region containing EGLN1 (a sliding window with 5 kb windows and 5 kb steps), calculated using the estimated allele frequency data of three populations (HNA, CLM, and CEU). Supplement Table 8: three statistical values (weighted FST, PBS, and PBS′) in an ~436.3 kb genomic region containing EGLN1 (a sliding window with 5 kb windows and 5 kb steps), calculated using the estimated allele frequency data of three populations (HNA, CLM, and JPT). Supplement Table 9: three statistical values (weighted FST, PBS, and PBS′) in an ~436.3 kb genomic region containing EGLN1 (a sliding window with 5 kb windows and 5 kb steps), calculated using the estimated allele frequency data of three populations (HNA, CLM, and YRI). Supplement Table 10: PBS′ in an ~436.3 kb genomic region located 1 Mb upstream of EGLN1 (a sliding window with 5 kb windows and 5 kb steps), calculated using the estimated allele frequency data for two population triplets (HNA, CLM, and JPT or CEU). Supplement Table 11: PBS′ in an ~436.3 kb genomic region located 1 Mb downstream of EGLN1 (a sliding window with 5 kb windows and 5 kb steps), calculated u [file 3436581.f1.pdf]

Table S1. Genotype data for *EGLN1* SNPs in each individual

Cohort	ID[a]	SNP1[b]	SNP2[b]	SNP3[b]	SNP4[b]	Cohort	ID[a]	SNP1[b]	SNP2[b]	SNP3[b]	SNP4[b]
La Paz	BLV001	T T	G A	C C	G G	El Alto	BLV051	T T	G G	C G	G A
La Paz	BLV002	T C	G A	C C	G A	El Alto	BLV052	T C	G A	C G	G A
La Paz	BLV003	T T	A A	C C	G G	El Alto	BLV053	T T	G A	C G	G A
La Paz	BLV004	T C	A A	C G	G A	El Alto	BLV054	T T	G G	C G	G A
La Paz	BLV005	T T	G G	C C	G G	El Alto	BLV055	T T	G G	C C	G G
La Paz	BLV006	T C	G A	C G	G A	El Alto	BLV056	T C	G A	C G	G A
La Paz	BLV007	T T	G G	C G	G A	El Alto	BLV057	T T	G G	C C	G G
La Paz	BLV008	T C	G A	C C	G A	El Alto	BLV058	T T	A A	C C	G G
La Paz	BLV009	T T	G G	C G	G A	El Alto	BLV059	T C	G A	C C	G A
La Paz	BLV010	T T	G A	C C	G A	El Alto	BLV060	T T	G G	C C	G A
La Paz	BLV011	T T	G A	C C	G A	El Alto	BLV061	T C	A A	C C	G A
La Paz	BLV012	T T	G G	C G	A A	El Alto	BLV062	T C	G A	C C	G A
La Paz	BLV013	T T	G G	C C	G G	El Alto	BLV063	T C	G A	C C	G A
La Paz	BLV014	C C	A A	G G	A A	El Alto	BLV064	T C	G A	C C	G A
La Paz	BLV015	T T	G A	C C	G A	El Alto	BLV065	T T	G G	C C	G G
La Paz	BLV016	T T	G A	C C	G G	El Alto	BLV066	C C	A A	C C	G A
La Paz	BLV017	T C	G A	C C	G A	El Alto	BLV067	T T	G A	C C	G G
La Paz	BLV018	T T	G G	C C	G G	El Alto	BLV068	T T	G G	C C	G A
La Paz	BLV019	T T	G G	C C	G G	El Alto	BLV069	T T	G G	C G	G A
La Paz	BLV020	C C	A A	C G	G A	El Alto	BLV070	T T	G G	C C	G G
La Paz	BLV021	T T	G A	C C	G G	El Alto	BLV071	T T	G G	C C	G G
La Paz	BLV022	T C	A A	C C	G A	El Alto	BLV072	T T	G G	C C	G G
La Paz	BLV023	T T	G A	C C	G A	El Alto	BLV073	T T	G G	C C	G G
La Paz	BLV024	T C	G A	C C	G A	El Alto	BLV074	T T	G G	C C	G G
La Paz	BLV025	T C	G A	C G	G A	El Alto	BLV075	T T	G A	C C	G G
La Paz	BLV026	C C	A A	C C	A A	El Alto	BLV077	T T	G G	C C	G G
La Paz	BLV027	T T	G G	C C	G G	El Alto	BLV078	T T	G A	C C	G G
La Paz	BLV028	C C	A A	C C	A A	El Alto	BLV079	T C	A A	C C	G G
La Paz	BLV029	T T	G G	C G	G A	El Alto	BLV080	T C	G A	C C	G A
La Paz	BLV030	T T	G G	C G	G A	El Alto	BLV081	T T	G A	C C	G G
La Paz	BLV031	T T	G G	C G	G A	El Alto	BLV082	T T	G G	C G	G A
La Paz	BLV034	T C	G A	C C	G A	El Alto	BLV083	T C	G A	G G	A A
La Paz	BLV035	T C	G A	C C	A A	El Alto	BLV084	C C	A A	C C	A A
La Paz	BLV036	T T	G A	C C	G G	El Alto	BLV085	T C	G A	C G	G A
La Paz	BLV037	T T	G G	C C	G A	El Alto	BLV086	T C	A A	C C	G A
La Paz	BLV038	T C	G A	C C	G A	El Alto	BLV087	T T	G G	C C	G G
La Paz	BLV039	T C	G A	C G	G A	El Alto	BLV089	T C	G A	C C	A A
La Paz	BLV040	T T	G A	C C	G G	El Alto	BLV090	C C	A A	C C	G A
La Paz	BLV041	T C	G A	C G	G A	El Alto	BLV091	T T	G G	C C	G G
La Paz	BLV042	T C	A A	C C	G A	El Alto	BLV092	T C	G A	C C	G A
La Paz	BLV043	C C	A A	C C	G A	El Alto	BLV093	T C	G A	C C	G A
La Paz	BLV044	T T	G A	C G	A A	El Alto	BLV094	T T	G G	C G	G A
La Paz	BLV045	T C	G A	C C	G A	El Alto	BLV095	T T	G G	C G	A A
La Paz	BLV046	T C	G A	C G	G A	El Alto	BLV096	T C	G A	C C	G A
La Paz	BLV047	T C	G A	C G	G A	El Alto	BLV097	T T	G G	C G	G A
La Paz	BLV048	T C	G A	C C	G G	El Alto	BLV098	T T	G G	C C	G G
La Paz	BLV049	T T	G G	C C	G G	El Alto	BLV099	T C	G A	C C	G A
La Paz	BLV050	T T	G G	C G	G A	El Alto	BLV100	T T	G G	C C	G G
						El Alto	BLV101	T C	G A	C G	A A
						El Alto	BLV102	T C	G A	G G	A A
						El Alto	BLV103	T T	G G	C C	G G

Abbreviation: SNP, single nucleotide polymorphism

[a] Individual ID

[b] SNP1, rs2486740; SNP2, rs508618; SNP3, rs12097901; and SNP4, rs1769792

Table S2. Allele frequencies of four SNPs (rs2486740, s508618, rs12097901, and rs1769792) around *EGLN1* in different human populations

	AFR							
**RefSNP ID**	**AFR_All**	**ACB[a]**	**ASW[a]**	**ESN[a]**	**GWD[a]**	**LWK[a]**	**MSL[a]**	**YRI[a]**
rs2486740	T: 0.738 (975)	T: 0.703 (135)	T: 0.664 (81)	T: 0.717 (142)	T: 0.743 (168)	T: 0.803 (159)	T: 0.776 (132)	T: 0.731 (158)
	C: 0.262 (347)	C: 0.297 (57)	C: 0.336 (41)	C: 0.283 (56)	C: 0.257 (58)	C: 0.197 (39)	C: 0.224 (38)	C: 0.269 (58)
rs508618	G: 0.190 (251)	G: 0.182 (35)	G: 0.238 (29)	G: 0.187 (37)	G: 0.133 (30)	G: 0.212 (42)	G: 0.188 (32)	G: 0.213 (46)
	A: 0.810 (1071)	A: 0.818 (157)	A: 0.762 (93)	A: 0.813 (161)	A: 0.867 (196)	A: 0.788 (156)	A: 0.812 (138)	A: 0.787 (170)
rs12097901	C: 0.663 (877)	C: 0.646 (124)	C: 0.713 (87)	C: 0.601 (119)	C: 0.659 (149)	C: 0.712 (141)	C: 0.665 (113)	C: 0.667 (144)
	G: 0.337 (445)	G: 0.354 (68)	G: 0.287 (35)	G: 0.399 (79)	G: 0.341 (77)	G: 0.288 (57)	G: 0.335 (57)	G: 0.333 (72)
rs1769792	G: 0.514 (679)	G: 0.469 (90)	G: 0.500 (61)	G: 0.530 (105)	G: 0.540 (122)	G: 0.495 (98)	G: 0.582 (99)	G: 0.481 (104)
	A: 0.486 (643)	A: 0.531 (102)	A: 0.500 (61)	A: 0.470 (93)	A: 0.460 (104)	A: 0.505 (100)	A: 0.418 (71)	A: 0.519 (112)

	AMR					
**RefSNP ID**	**AMR_All**	**BLV**	**CLM[a]**	**MXL[a]**	**PEL[a]**	**PUR[a]**
rs2486740	T: 0.539 (481)	T: 0.732 (145)	T: 0.410 (77)	T: 0.500 (64)	T: 0.653 (111)	T: 0.404 (84)
	C: 0.461 (411)	C: 0.268 (53)	C: 0.590 (111)	C: 0.500 (64)	C: 0.347 (59)	C: 0.596 (124)
rs508618	G: 0.371 (331)	G: 0.606 (120)	G: 0.191 (36)	G: 0.273 (35)	G: 0.559 (95)	G: 0.216 (45)
	A: 0.629 (561)	A: 0.394 (78)	A: 0.809 (152)	A: 0.727 (93)	A: 0.441 (75)	A: 0.784 (163)
rs12097901	C: 0.865 (772)	C: 0.828 (164)	C: 0.862 (162)	C: 0.883 (113)	C: 0.865 (147)	C: 0.894 (186)
	G: 0.135 (120)	G: 0.172 (34)	G: 0.138 (26)	G: 0.117 (15)	G: 0.135 (23)	G: 0.106 (22)
rs1769792	A: 0.571 (509)	G: 0.606 (120)	G: 0.309 (58)	G: 0.352 (45)	G: 0.535 (91)	G: 0.332 (69)
	G: 0.429 (383)	A: 0.394 (78)	A: 0.691 (130)	A: 0.648 (83)	A: 0.465 (79)	A: 0.668 (139)

	EAS					
**RefSNP ID**	**EAS_All**	**CDX[a]**	**CHB[a]**	**CHS[a]**	**JPT[a]**	**KHV[a]**
rs2486740	T: 0.467 (471)	T: 0.548 (102)	T: 0.442 (91)	T: 0.448 (94)	T: 0.462 (96)	T: 0.444 (88)
	C: 0.533 (537)	C: 0.452 (84)	C: 0.558 (115)	C: 0.552 (116)	C: 0.538 (112)	C: 0.556 (110)
rs508618	G: 0.165 (166)	G: 0.220 (41)	G: 0.112 (23)	G: 0.152 (32)	G: 0.173 (36)	G: 0.172 (34)
	A: 0.835 (842)	A: 0.780 (145)	A: 0.888 (183)	A: 0.848 (178)	A: 0.827 (172)	A: 0.828 (164)
rs12097901	C: 0.541 (545)	C: 0.575 (107)	C: 0.515 (106)	C: 0.543 (114)	C: 0.529 (110)	C: 0.545 (108)
	G: 0.459 (463)	G: 0.425 (79)	G: 0.485 (100)	G: 0.457 (96)	G: 0.471 (98)	G: 0.455 (90)
rs1769792	G: 0.033 (33)	G: 0.043 (8)	G: 0.029 (6)	G: 0.029 (6)	G: 0.058 (12)	G: 0.005 (1)
	A: 0.967 (975)	A: 0.957 (178)	A: 0.971 (200)	A: 0.971 (204)	A: 0.942 (196)	A: 0.995 (197)

	SAS					
**RefSNP ID**	**SAS_All**	**BEB[a]**	**GIH[a]**	**ITU[a]**	**PJL[a]**	**STU[a]**
rs2486740	T: 0.427 (418)	T: 0.424 (73)	T: 0.296 (61)	T: 0.426 (87)	T: 0.516 (99)	T: 0.480 (98)
	C: 0.573 (560)	C: 0.576 (99)	C: 0.704 (145)	C: 0.574 (117)	C: 0.484 (93)	C: 0.520 (106)
rs508618	G: 0.213 (208)	G: 0.203 (35)	G: 0.146 (30)	G: 0.245 (50)	G: 0.224 (43)	G: 0.245 (50)
	A: 0.787 (770)	A: 0.797 (137)	A: 0.854 (176)	A: 0.755 (154)	A: 0.776 (149)	A: 0.755 (154)
rs12097901	C: 0.744 (728)	C: 0.738 (127)	C: 0.845 (174)	C: 0.711 (145)	C: 0.729 (140)	C: 0.696 (142)
	G: 0.256 (250)	G: 0.262 (45)	G: 0.155 (32)	G: 0.289 (59)	G: 0.271 (52)	G: 0.304 (62)
rs1769792	G: 0.102 (100)	G: 0.099 (17)	G: 0.117 (24)	G: 0.069 (14)	G: 0.161 (31)	G: 0.069 (14)
	A: 0.898 (878)	A: 0.901 (155)	A: 0.883 (182)	A: 0.931 (190)	A: 0.839 (161)	A: 0.931 (190)
	**EUR**					
**RefSNP ID**	**EUR_All**	**CEU[a]**	**FIN[a]**	**GBR[a]**	**IBS[a]**	**TSI[a]**
rs2486740	T: 0.338 (340)	T: 0.379 (75)	T: 0.283 (56)	T: 0.308 (56)	T: 0.360 (77)	T: 0.355 (76)
	C: 0.662 (666)	C: 0.621 (123)	C: 0.717 (142)	C: 0.692 (126)	C: 0.640 (137)	C: 0.645 (138)
rs508618	G: 0.168 (169)	G: 0.207 (41)	G: 0.187 (37)	G: 0.148 (27)	G: 0.136 (29)	G: 0.164 (35)
	A: 0.832 (837)	A: 0.793 (157)	A: 0.813 (161)	A: 0.852 (155)	A: 0.864 (185)	A: 0.836 (179)
rs12097901	C: 0.940 (946)	C: 0.924 (183)	C: 0.924 (183)	C: 0.962 (175)	C: 0.949 (203)	C: 0.944 (202)
	G: 0.060 (60)	G: 0.076 (15)	G: 0.076 (15)	G: 0.038 (7)	G: 0.051 (11)	G: 0.056 (12)
rs1769792	G: 0.251 (253)	G: 0.278 (55)	G: 0.187 (37)	G: 0.242 (44)	G: 0.290 (62)	G: 0.257 (55)
	A: 0.749 (753)	A: 0.722 (143)	A: 0.813 (161)	A: 0.758 (138)	A: 0.710 (152)	A: 0.743 (159)

Values indicate allele frequencies, and the observed numbers are indicated in parentheses.

AFR, African. ACB: African Caribbeans in Barbados. ASW: Americans of African Ancestry in SW USA. ESN: Esan in Nigeria. GWD: Gambian in Western Divisions in the Gambia. LWK: Luhya in Webuye, Kenya. MSL: Mende in Sierra Leone. YRI: Yoruba in Ibadan, Nigeria. AMR, American. BLV: Bolivian from La Paz and El Alto, Bolivia. CLM: Colombians from Medellin, Colombia. MXL: Mexican Ancestry from Los Angeles USA. PEL: Peruvians from Lima, Peru. PUR: Puerto Ricans from Puerto Rico. EAS, East Asian. CDX: Chinese Dai in Xishuangbanna, China. CHB: Han Chinese in Beijing, China. CHS: Southern Han Chinese. JPT: Japanese in Tokyo, Japan. KHV: Kinh in Ho Chi Minh City, Vietnam. SAS, South Asian. BEB: Bengali from Bangladesh. GIH: Gujarati Indian from Houston, Texas. ITU: Indian Telugu from the UK. PJL: Punjabi from Lahore, Pakistan. STU: Sri Lankan Tamil from the UK. EUR, European. CEU: Utah Residents with Northern and Western European Ancestry. FIN: Finnish in Finland. GBR: British in England and Scotland. IBS: Iberian Population in Spain. TSI: Toscani in Italia

[a]Allele frequency obtained from the 1000 Genomes Project database

Table S3. Linkage disequilibrium (r^2 below the diagonal and D′ above the diagonal) of four SNPs around *EGLN1* in five ethnic groups from the 1000 Genomes Project

		rs2486740	rs508618	rs12097901	rs1769792
**AMR**			D′		
rs2486740	r^2		1.000	0.015	0.816
rs508618		0.465		0.044	0.405
rs12097901		0.000	0.000		0.755
rs1769792		0.433	0.117	0.049	
**EAS**			D′		
rs2486740	r^2		1.000	0.619	0.772
rs508618		0.225		0.376	0.637
rs12097901		0.371	0.033		1.000
rs1769792		0.023	0.070	0.029	
**SAS**			D′		
rs2486740	r^2		0.958	0.609	0.808
rs508618		0.332		0.191	0.416
rs12097901		0.171	0.003		0.922
rs1769792		0.100	0.073	0.033	
**EUR**			D′		
rs2486740	r^2		1.000	0.396	0.755
rs508618		0.396		0.802	0.383
rs12097901		0.019	0.008		0.404
rs1769792		0.375	0.088	0.003	
**AFR**			D′		
rs2486740	r^2		0.985	0.803	0.495
rs508618		0.081		0.508	0.387
rs12097901		0.116	0.119		0.414
rs1769792		0.092	0.037	0.092	

Abbreviations: SNP, single nucleotide polymorphism. AMR, American. EAS, East Asian. SAS, South Asian. EUR, European. AFR, African

Table S4. Statistical tests for the association between *EGLN1* genotypes and hematological traits

Phenotype	RefSNP ID	Position	Cohort	Welch's *t*-test (Dominant[e])		Linear regression (Additive[e])	
				t statistic	*p* value	Estimate	*p* value
Hb levels[a]	rs2486740	1: 231517552[b]	Entire[d]	1.672	0.098	-0.083	0.076
		1: 231381806[c]	La Paz	1.855	0.071	-0.166	0.019[*]
			El Alto	0.538	0.593	-0.008	0.897
	rs508618	1: 231532312[b]	Entire[d]	1.539	0.128	-0.101	0.048[*]
		1: 231396566[c]	La Paz	2.190	0.035[*]	-0.188	0.009[**]
			El Alto	0.422	0.675	-0.022	0.765
	rs12097901	1: 231557255[b]	Entire[d]	0.799	0.428	-0.037	0.344
		1: 231421509[c]	La Paz	0.602	0.552	-0.049	0.390
			El Alto	0.496	0.625	-0.026	0.637
	rs1769792	1: 231598618[b]	Entire[d]	2.487	0.015[*]	-0.077	0.104
		1: 231462872[c]	La Paz	2.854	0.008[**]	-0.133	0.041[*]
			El Alto	1.051	0.299	-0.025	0.718
SpO_2[a]	rs2486740	1: 231517552[b]	Entire[d]	0.779	0.438	-0.007	0.779
		1: 231381806[c]	La Paz	-0.397	0.693	0.029	0.488
			El Alto	1.754	0.086	-0.051	0.139
	rs508618	1: 231532312[b]	Entire[d]	-0.015	0.988	-0.013	0.648
		1: 231396566[c]	La Paz	-0.726	0.477	0.028	0.517
			El Alto	1.375	0.176	-0.076	0.055
	rs12097901	1: 231557255[b]	Entire[d]	-0.211	0.834	0.006	0.761
		1: 231421509[c]	La Paz	-1.470	0.150	0.055	0.091
			El Alto	1.470	0.155	-0.041	0.184
	rs1769792	1: 231598618[b]	Entire[d]	-0.535	0.595	0.017	0.514
		1: 231462872[c]	La Paz	-1.571	0.136	0.089	0.016[*]
			El Alto	1.510	0.138	-0.058	0.121
Heart rate	rs2486740	1: 231517552[b]	Entire[d]	1.665	0.099	-0.008	0.142
		1: 231381806[c]	La Paz	1.597	0.119	-0.009	0.231
			El Alto	0.798	0.429	-0.009	0.293
	rs508618	1: 231532312[b]	Entire[d]	0.653	0.516	5.1×10^{-5}	0.993
		1: 231396566[c]	La Paz	1.007	0.328	-3.5×10^{-3}	0.650
			El Alto	0.169	0.866	1.5×10^{-3}	0.883
	rs12097901	1: 231557255[b]	Entire[d]	-0.756	0.453	3.3×10^{-3}	0.476
		1: 231421509[c]	La Paz	1.023	0.313	-0.007	0.251
			El Alto	-2.353	0.028[*]	0.018	0.015[*]
	rs1769792	1: 231598618[b]	Entire[d]	0.780	0.439	-3.1×10^{-3}	0.569
		1: 231462872[c]	La Paz	0.823	0.422	-0.008	0.239
			El Alto	0.553	0.584	1.8×10^{-3}	0.845
SBP	rs2486740	1: 231517552[b]	Entire[d]	0.249	0.804	-1.2×10^{-3}	0.825
		1: 231381806[c]	La Paz	0.242	0.810	-4.7×10^{-3}	0.589
			El Alto	-0.089	0.930	4.3×10^{-3}	0.564
	rs508618	1: 231532312[b]	Entire[d]	-1.199	0.234	0.006	0.338
		1: 231396566[c]	La Paz	-1.742	0.093	0.005	0.544
			El Alto	-0.923	0.360	0.013	0.138
	rs12097901	1: 231557255[b]	Entire[d]	1.203	0.233	-4.6×10^{-3}	0.300
		1: 231421509[c]	La Paz	1.006	0.320	-0.007	0.315
			El Alto	0.375	0.711	-1.9×10^{-3}	0.772
	rs1769792	1: 231598618[b]	Entire[d]	1.742	0.087	-0.007	0.174
		1: 231462872[c]	La Paz	1.447	0.169	-0.011	0.141
			El Alto	0.441	0.662	1.4×10^{-3}	0.868

DBP	rs2486740	1: 231517552[b]	Entire[d]	0.425	0.672	2.0×10^{-3}	0.787
		1: 231381806[c]	La Paz	0.374	0.710	-4.1×10^{-5}	0.998
			El Alto	-0.066	0.948	2.4×10^{-3}	0.831
	rs508618	1: 231532312[b]	Entire[d]	0.724	0.472	-4.7×10^{-3}	0.573
		1: 231396566[c]	La Paz	-0.651	0.521	0.009	0.561
			El Alto	0.227	0.822	-9.5×10^{-4}	0.942
	rs12097901	1: 231557255[b]	Entire[d]	0.007	0.994	1.4×10^{-3}	0.827
		1: 231421509[c]	La Paz	0.011	0.992	-1.5×10^{-3}	0.898
			El Alto	-0.725	0.476	0.009	0.357
	rs1769792	1: 231598618[b]	Entire[d]	0.635	0.527	1.9×10^{-3}	0.803
		1: 231462872[c]	La Paz	0.250	0.806	3.5×10^{-3}	0.798
			El Alto	-0.353	0.726	0.012	0.312

Abbreviations: Hb, hemoglobin. SpO_2, percutaneous arterial oxygen saturation. SBP, systolic blood pressure. DBP, diastolic blood pressure

[a] Estimated value obtained by using a noninvasive method

[b] Chromosomal position in NCBI build GRCh37

[c] Chromosomal position in NCBI build GRCh38

[d] Combined cohort of La Paz and El Alto cohorts

[e] Genetic inheritance model

[*] $p < 0.05$

[**] $p < 0.01$

Table S5. Hematological characteristics of 99 Bolivian highlanders with different genotypes of four SNPs around *EGLN1*

RefSNP ID	Position	Genotype	Cohort	*N*[d]	Hb (g/dL)[e, f, g]	SpO_2 (%)[e, f]	HR (bpm)[e]	SBP (mmHg)[e]	DBP (mmHg)[e]
rs2486740	1: 231517552[a]	TT	Entire[c]	54	14.6 ± 1.4	90.6 ± 2.6	78.8 ± 13.6	113.9 ± 12.8	66.2 ± 9.0
	1: 231381806[b]		La Paz	25	14.7 ± 1.2	91.2 ± 2.7	82.0 ± 16.3	110.2 ± 12.7	61.2 ± 6.9
			El Alto	29	15.0 ± 1.1	89.3 ± 3.2	69.0 ± 11.1	126.0 ± 11.0	72.3 ± 8.3
		TC	Entire[c]	37	14.2 ± 1.4	90.1 ± 2.5	75.0 ± 9.1	113.3 ± 11.2	65.3 ± 8.4
			La Paz	18	14.2 ± 1.6	91.2 ± 2.1	75.6 ± 8.2	110.3 ± 10.9	59.9 ± 6.2
			El Alto	19	14.2 ± 1.3	88.9 ± 2.3	74.5 ± 10.1	116.2 ± 11.0	70.3 ± 7.0
		CC	Entire[c]	8	13.8 ± 1.3	91.1 ± 2.7	74.9 ± 9.3	113.4 ± 13.8	66.3 ± 8.7
			La Paz	5	13.1 ± 0.8	92.2 ± 1.9	78.4 ± 7.0	105.8 ± 9.1	62.6 ± 7.3
			El Alto	3	14.5 ± 1.6	90.2 ± 2.5	76.0 ± 10.3	117.2 ± 12.3	70.4 ± 8.4
rs508618	1: 231532312[a]	GG	Entire[c]	37	14.7 ± 1.4	90.5 ± 2.6	78.1 ± 13.1	111.8 ± 11.5	66.7 ± 9.0
	1: 231396566[b]		La Paz	14	14.9 ± 1.0	90.9 ± 2.8	82.8 ± 17.1	105.4 ± 10.9	59.9 ± 6.6
			El Alto	23	14.4 ± 1.3	88.0 ± 2.7	77.0 ± 14.0	124.4 ± 14.3	71.0 ± 6.6
		GA	Entire[c]	46	14.3 ± 1.4	90.7 ± 2.4	75.4 ± 11.0	114.9 ± 12.3	65.3 ± 8.7
			La Paz	25	14.4 ± 1.5	91.5 ± 2.3	76.4 ± 11.7	113.3 ± 12.3	61.2 ± 6.8
			El Alto	21	14.3 ± 1.2	89.7 ± 2.2	74.1 ± 10.3	116.8 ± 12.2	70.0 ± 8.4
		AA	Entire[c]	16	13.8 ± 1.3	89.9 ± 2.9	79.6 ± 11.1	114.5 ± 14.0	65.6 ± 8.1
			La Paz	9	13.4 ± 1.1	91.4 ± 2.2	81.6 ± 8.6	106.8 ± 7.6	61.4 ± 6.7
			El Alto	7	14.5 ± 1.7	90.2 ± 2.6	75.3 ± 9.3	115.7 ± 10.2	70.8 ± 7.7
rs12097901	1: 231557255[a]	CC	Entire[c]	68	14.5 ± 1.4	90.4 ± 2.5	76.5 ± 12.1	114.6 ± 12.8	65.9 ± 8.5
	1: 231421509[b]		La Paz	31	14.4 ± 1.4	90.9 ± 2.5	80.6 ± 13.6	110.9 ± 12.8	60.9 ± 6.9
			El Alto	37	14.5 ± 1.4	90.0 ± 2.5	73.0 ± 9.6	117.7 ± 12.1	70.0 ± 7.5
		CG	Entire[c]	28	14.2 ± 1.6	90.5 ± 2.7	78.4 ± 11.7	111.5 ± 11.1	65.5 ± 9.1
			La Paz	16	14.3 ± 1.4	91.8 ± 2.0	77.3 ± 12.4	108.1 ± 9.2	61.1 ± 6.4
			El Alto	12	14.2 ± 1.8	88.8 ± 2.7	79.8 ± 11.0	116.2 ± 12.0	71.3 ± 9.0
		GG	Entire[c]	3	13.8 ± 0.9	91.0 ± 3.5	78.7 ± 10.2	112.3 ± 10.0	69.3 ± 9.8
			La Paz	1	12.8 ± NA	95.0 ± NA	67.0 ± NA	102.0 ± NA	58.0 ± NA
			El Alto	2	14.3 ± 0.4	89.0 ± 0.0	84.5 ± 2.1	117.5 ± 6.4	75.0 ± 0.0
rs1769792	1: 231598618[a]	GG	Entire[c]	33	14.8 ± 1.2	90.3 ± 2.5	78.5 ± 13.4	116.8 ± 13.6	66.6 ± 8.1
	1: 231462872[b]		La Paz	13	15.1 ± 1.0	90.2 ± 3.1	82.2 ± 16.4	114.7 ± 15.9	61.3 ± 7.1
			El Alto	20	14.8 ± 0.8	88.7 ± 1.0	80.0 ± 10.4	118.8 ± 11.7	75.2 ± 8.5
		GA	Entire[c]	54	14.1 ± 1.5	90.5 ± 2.7	76.2 ± 11.3	111.7 ± 10.9	64.7 ± 8.5

	La Paz	29	14.1 ± 1.5	91.4 ± 1.9	78.9 ± 12.1	107.9 ± 8.5	60.3 ± 6.0
	El Alto	25	14.1 ± 1.6	89.4 ± 3.0	73.0 ± 9.5	116.2 ± 11.8	69.8 ± 8.1
AA	Entire[c]	12	14.4 ± 1.0	90.8 ± 2.7	77.3 ± 10.3	113.6 ± 13.0	69.1 ± 10.4
	La Paz	6	14.0 ± 1.2	93.0 ± 1.8	74.7 ± 10.5	108.3 ± 13.0	63.0 ± 8.8
	El Alto	6	14.7 ± 1.3	90.3 ± 2.0	76.1 ± 10.8	118.3 ± 12.1	70.1 ± 6.8

Abbreviations: SNP, single nucleotide polymorphism. Hb, hemoglobin level. SpO_2, percutaneous arterial oxygen saturation. HR, heart rate. SBP, systolic blood pressure. DBP, diastolic blood pressure. NA, not applicable

[a] Chromosomal position in NCBI build GRCh37

[b] Chromosomal position in NCBI build GRCh38

[c] Combined cohort of La Paz and El Alto cohorts

[d] The number of subjects

[e] Quantitative data are presented as the mean ± standard deviation of the mean

[f] Estimated value obtained by using a noninvasive method

[g] One individual was removed due to a lack of Hb measurement

Table S6. Observed number of haplotypes for four SNPs (rs2486740-s508618-rs12097901-rs1769792) around *EGLN1* in different human populations

		Haplotype													
Ethnic group	Population	CACA	TACG	TAGA	TGCG	TGGA	TGCA	CAGA	CACG	TACA	TAGG	TGGG	CGCA	CAGG	Total
AMR	BLV	29	19	2	84	18	15	10	13	3	2	2	1	0	198
	CLM[a]	95	34	5	14	7	12	10	6	1	1	3	0	0	188
	MXL[a]	51	24	2	16	1	18	10	3	1	2	0	0	0	128
	PEL[a]	43	14	1	74	8	13	13	2	1	0	0	0	1	170
	PUR[a]	100	30	5	25	5	14	11	13	4	0	1	0	0	208
EAS	CDX[a]	71	0	46	8	20	13	13	0	15	0	0	0	0	186
	CHB[a]	88	3	57	2	17	4	26	1	8	0	0	0	0	206
	CHS[a]	98	0	56	6	22	4	18	0	6	0	0	0	0	210
	JPT[a]	85	3	48	6	26	4	24	3	9	0	0	0	0	208
	KHV[a]	97	0	52	1	25	8	13	0	2	0	0	0	0	198
SAS	BEB[a]	89	4	32	10	6	19	7	3	2	0	0	0	0	172
	GIH[a]	126	7	13	9	6	13	11	6	11	2	0	2	0	206
	ITU[a]	99	3	32	10	13	24	14	1	5	0	0	3	0	204
	PJL[a]	84	15	36	15	8	20	8	1	5	0	0	0	0	192
	STU[a]	90	4	36	10	10	30	16	0	8	0	0	0	0	204
EUR	CEU[a]	105	21	7	20	0	21	6	12	4	2	0	0	0	198
	FIN[a]	131	9	5	21	0	16	7	4	2	3	0	0	0	198
	GBR[a]	116	19	4	18	0	9	3	7	6	0	0	0	0	182
	IBS[a]	120	35	3	14	1	14	5	11	9	1	0	0	1	214
	TSI[a]	130	29	7	18	1	16	2	6	3	2	0	0	0	214
AFR	ACB[a]	42	51	24	7	19	2	4	11	11	14	7	0	0	192
	ASW[a]	26	28	16	17	9	1	4	10	4	5	1	1	0	122
	ESN[a]	33	54	26	4	25	2	2	21	5	20	6	0	0	198
	GWD[a]	37	84	30	4	24	2	3	14	8	16	0	0	4	226
	LWK[a]	32	68	16	8	24	10	1	6	17	16	0	0	0	198
	MSL[a]	29	59	11	6	17	5	2	7	7	23	4	0	0	170
	YRI[a]	39	61	25	9	27	4	2	16	15	11	6	0	1	216
	Total	2085	678	597	436	339	313	245	177	172	120	30	7	7	5206

AMR, American. BLV: Bolivian from La Paz and El Alto, Bolivia. CLM: Colombians from Medellin, Colombia. MXL: Mexican Ancestry from Los Angeles USA. PEL: Peruvians from Lima, Peru. PUR: Puerto Ricans from Puerto Rico. EAS, East Asian. CDX: Chinese Dai in Xishuangbanna, China. CHB: Han Chinese in Beijing, China. CHS: Southern Han Chinese. JPT: Japanese in Tokyo, Japan. KHV: Kinh in Ho Chi Minh City, Vietnam. SAS, South Asian. BEB: Bengali from Bangladesh. GIH: Gujarati Indian from Houston, Texas. ITU: Indian Telugu from the UK. PJL: Punjabi from Lahore, Pakistan. STU: Sri Lankan Tamil from

the UK. EUR, European. CEU: Utah Residents with Northern and Western European Ancestry. FIN: Finnish in Finland. GBR: British in England and Scotland. IBS: Iberian Population in Spain. TSI: Toscani in Italia. AFR, African. ACB: African Caribbeans in Barbados. ASW: Americans of African Ancestry in SW USA. ESN: Esan in Nigeria. GWD: Gambian in Western Divisions in the Gambia. LWK: Luhya in Webuye, Kenya. MSL: Mende in Sierra Leone. YRI: Yoruba in Ibadan, Nigeria

[a]Observed number of haplotypes estimated using the variant call format (VCF) data in the 1000 Genomes Project database

Table S7. Three statistical values (weighted F_{ST}, PBS, and PBS′) in a ~436.3 kb genomic region containing *EGLN1* (a sliding window with 5 kb windows and 5 kb steps), calculated using the estimated allele frequency data of three populations (HNA, CLM, and CEU)

Position[a]	F_{ST} CEU-CLM	F_{ST} CEU-HNA	F_{ST} CLM-HNA	PBS_{CEU}	PBS_{CLM}	PBS_{HNA}	PBS'_{CEU}	PBS'_{CLM}	PBS'_{HNA}
1:231367500	0.0028	0.1342	0.1513	-0.0086	0.0114	0.1527	-0.0074	0.0098	0.1321
1:231372500	0.0111	0.0463	0.0199	0.0192	-0.0081	0.0282	0.0185	-0.0077	0.0271
1:231377500	-0.0077	0.0940	0.0793	0.0042	-0.0119	0.0945	0.0038	-0.0109	0.0870
1:231382500	0.0096	0.0487	0.0427	0.0080	0.0016	0.0420	0.0076	0.0015	0.0399
1:231387500	0.0125	0.0393	0.0573	-0.0031	0.0157	0.0433	-0.0030	0.0149	0.0410
1:231392500	0.0102	0.1188	0.1350	-0.0042	0.0144	0.1306	-0.0036	0.0127	0.1145
1:231397500	0.0170	0.0387	0.0683	-0.0070	0.0242	0.0465	-0.0066	0.0227	0.0437
1:231402500	-0.0032	0.0720	0.0960	-0.0147	0.0115	0.0894	-0.0135	0.0106	0.0823
1:231407500	0.0093	0.0283	0.0408	-0.0018	0.0111	0.0305	-0.0017	0.0107	0.0293
1:231412500	0.0077	0.0600	0.0631	0.0022	0.0055	0.0597	0.0020	0.0052	0.0559
1:231417500	0.0178	0.0505	0.0892	-0.0119	0.0298	0.0637	-0.0110	0.0275	0.0589
1:231422500	0.0072	0.0476	0.0459	0.0045	0.0027	0.0443	0.0043	0.0026	0.0421
1:231427500	0.0083	0.1180	0.0935	0.0179	-0.0095	0.1077	0.0160	-0.0085	0.0965
1:231432500	0.0213	0.0406	0.0177	0.0226	-0.0010	0.0189	0.0217	-0.0010	0.0181
1:231437500	0.0423	0.0372	0.0965	-0.0102	0.0534	0.0481	-0.0093	0.0489	0.0441
1:231442500	0.0346	0.1384	0.0744	0.0535	-0.0183	0.0955	0.0473	-0.0161	0.0845
1:231447500	0.0531	0.0193	0.0141	0.0299	0.0246	-0.0104	0.0287	0.0236	-0.0100
1:231452500	0.0164	0.0230	0.0220	0.0088	0.0078	0.0145	0.0085	0.0075	0.0140
1:231457500	-0.0003	0.1015	0.0871	0.0078	-0.0081	0.0992	0.0071	-0.0073	0.0903
1:231462500	0.0017	0.1309	0.1734	-0.0242	0.0259	0.1646	-0.0208	0.0222	0.1411
1:231467500	0.0001	0.1226	0.1410	-0.0106	0.0107	0.1413	-0.0093	0.0093	0.1238
1:231472500	-0.0006	0.1062	0.1205	-0.0083	0.0078	0.1206	-0.0075	0.0070	0.1077
1:231477500	0.0031	0.1118	0.1735	-0.0345	0.0375	0.1530	-0.0298	0.0325	0.1323
1:231482500	0.0037	0.0954	0.1325	-0.0191	0.0228	0.1194	-0.0170	0.0203	0.1063
1:231487500	0.0096	0.1232	0.1754	-0.0259	0.0355	0.1574	-0.0222	0.0304	0.1348
1:231492500	0.0032	0.1306	0.1767	-0.0256	0.0288	0.1656	-0.0219	0.0247	0.1417
1:231497500	-0.0041	0.1302	0.1626	-0.0210	0.0169	0.1606	-0.0182	0.0146	0.1388
1:231502500	-0.0011	0.1835	0.1987	-0.0099	0.0089	0.2126	-0.0082	0.0073	0.1755
1:231507500	0.0053	0.1542	0.1801	-0.0129	0.0182	0.1804	-0.0109	0.0154	0.1521
1:231512500	0.0011	0.1474	0.1934	-0.0272	0.0283	0.1867	-0.0229	0.0238	0.1572
1:231517500	-0.0022	0.1523	0.1922	-0.0252	0.0230	0.1904	-0.0212	0.0194	0.1603
1:231522500	-0.0017	0.1315	0.1644	-0.0202	0.0184	0.1612	-0.0174	0.0159	0.1390
1:231527500	0.0060	0.1365	0.1566	-0.0088	0.0148	0.1555	-0.0076	0.0128	0.1339
1:231532500	0.0007	0.1450	0.1855	-0.0239	0.0247	0.1806	-0.0203	0.0209	0.1528
1:231537500	0.0044	0.1435	0.2010	-0.0325	0.0369	0.1874	-0.0273	0.0310	0.1573
1:231542500	0.0622	0.1243	0.1841	-0.0033	0.0675	0.1360	-0.0027	0.0562	0.1133
1:231547500	0.0647	0.1953	0.2276	0.0130	0.0540	0.2043	0.0102	0.0424	0.1607
1:231552500	0.0748	0.0964	0.1511	0.0076	0.0701	0.0938	0.0065	0.0598	0.0800
1:231557500	0.0581	0.1575	0.0737	0.0773	-0.0175	0.0941	0.0670	-0.0151	0.0815
1:231562500	0.1020	0.3151	0.1632	0.1539	-0.0464	0.2245	0.1155	-0.0348	0.1685
1:231567500	0.0375	0.2291	0.0804	0.1073	-0.0691	0.1529	0.0901	-0.0580	0.1283
1:231572500	0.0572	0.1908	0.0700	0.0990	-0.0402	0.1127	0.0845	-0.0343	0.0962
1:231577500	0.1294	0.3791	0.1346	0.2353	-0.0967	0.2413	0.1705	-0.0701	0.1749
1:231582500	0.1007	0.3650	0.1340	0.2082	-0.1021	0.2460	0.1540	-0.0755	0.1819
1:231587500	0.0908	0.3708	0.2197	0.1552	-0.0600	0.3080	0.1106	-0.0427	0.2195
1:231592500	0.0875	0.3414	0.1754	0.1582	-0.0666	0.2594	0.1171	-0.0493	0.1920
1:231597500	0.0933	0.3445	0.1347	0.1878	-0.0899	0.2346	0.1409	-0.0675	0.1760
1:231602500	0.0488	0.1909	0.1107	0.0723	-0.0222	0.1395	0.0607	-0.0186	0.1173
1:231607500	0.0662	0.2009	0.0685	0.1109	-0.0424	0.1134	0.0938	-0.0359	0.0959
1:231612500	0.0218	0.3122	0.2012	0.0858	-0.0638	0.2885	0.0655	-0.0487	0.2201

1:231617500	0.0242	0.0353	0.0208	0.0197	0.0048	0.0162	0.0189	0.0046	0.0156
1:231622500	0.0017	0.0478	0.0870	-0.0202	0.0218	0.0692	-0.0188	0.0204	0.0646
1:231627500	0.1118	0.1879	0.0575	0.1337	-0.0152	0.0744	0.1121	-0.0127	0.0623
1:231632500	0.0433	0.2673	0.1468	0.0982	-0.0540	0.2127	0.0781	-0.0429	0.1692
1:231637500	0.0364	0.3064	0.1612	0.1136	-0.0765	0.2523	0.0881	-0.0593	0.1957
1:231642500	0.1020	0.1001	0.0142	0.0994	0.0082	0.0061	0.0892	0.0073	0.0055
1:231647500	0.0264	0.1722	0.0508	0.0818	-0.0551	0.1072	0.0722	-0.0485	0.0945
1:231652500	0.1029	0.2521	0.0735	0.1614	-0.0528	0.1292	0.1304	-0.0427	0.1044
1:231657500	0.0537	0.0834	0.0966	0.0203	0.0349	0.0667	0.0181	0.0311	0.0595
1:231662500	0.1229	0.2103	0.1097	0.1255	0.0056	0.1105	0.1011	0.0045	0.0890
1:231667500	0.0352	0.0758	0.0333	0.0404	-0.0045	0.0384	0.0376	-0.0042	0.0358
1:231672500	0.0500	0.2446	0.1476	0.0861	-0.0348	0.1945	0.0691	-0.0279	0.1561
1:231677500	0.0598	0.2844	0.0998	0.1456	-0.0839	0.1890	0.1164	-0.0671	0.1511
1:231682500	0.0661	0.1628	0.0941	0.0736	-0.0052	0.1040	0.0628	-0.0044	0.0887
1:231687500	0.0816	0.2123	0.0907	0.1143	-0.0292	0.1243	0.0945	-0.0242	0.1028
1:231692500	0.0572	0.3238	0.1279	0.1567	-0.0978	0.2346	0.1211	-0.0756	0.1814
1:231697500	0.0490	0.2166	0.1557	0.0626	-0.0123	0.1815	0.0508	-0.0100	0.1474
1:231702500	0.0787	0.1944	0.0810	0.1068	-0.0248	0.1094	0.0897	-0.0209	0.0918
1:231707500	0.0299	0.2061	0.1041	0.0756	-0.0452	0.1552	0.0638	-0.0381	0.1309
1:231712500	0.0214	0.1794	0.1173	0.0473	-0.0256	0.1504	0.0403	-0.0219	0.1283
1:231717500	0.0088	0.0740	0.0624	0.0107	-0.0018	0.0662	0.0099	-0.0017	0.0615
1:231722500	0.0490	0.1078	0.1375	0.0082	0.0420	0.1059	0.0071	0.0364	0.0916
1:231727500	0.0656	0.1219	0.0624	0.0667	0.0011	0.0633	0.0590	0.0010	0.0560
1:231732500	0.1830	0.0954	0.2194	0.0274	0.1748	0.0729	0.0215	0.1371	0.0572
1:231737500	0.2063	0.0518	0.2354	0.0079	0.2231	0.0453	0.0062	0.1748	0.0355
1:231742500	0.0839	0.0765	0.0381	0.0642	0.0235	0.0153	0.0582	0.0213	0.0139
1:231747500	0.2156	0.0929	0.0226	0.1587	0.0841	-0.0612	0.1343	0.0712	-0.0518
1:231752500	0.1433	0.0707	0.0343	0.0965	0.0581	-0.0232	0.0853	0.0514	-0.0205
1:231757500	0.1676	0.1074	0.0394	0.1284	0.0550	-0.0148	0.1099	0.0471	-0.0127
1:231762500	0.0990	0.0490	0.0169	0.0688	0.0355	-0.0185	0.0633	0.0327	-0.0170
1:231767500	0.1183	0.0600	0.0252	0.0811	0.0447	-0.0192	0.0733	0.0404	-0.0174
1:231772500	0.2024	0.0823	0.0179	0.1470	0.0792	-0.0611	0.1262	0.0680	-0.0525
1:231777500	0.1662	0.0763	0.0168	0.1221	0.0597	-0.0428	0.1072	0.0524	-0.0375
1:231782500	0.1026	0.0635	0.0106	0.0815	0.0267	-0.0160	0.0747	0.0244	-0.0146
1:231787500	0.0369	0.0541	0.1173	-0.0158	0.0534	0.0714	-0.0142	0.0482	0.0643
1:231792500	0.1251	0.0610	0.0224	0.0870	0.0467	-0.0240	0.0784	0.0421	-0.0217
1:231797500	0.0389	0.0347	0.0117	0.0316	0.0081	0.0037	0.0303	0.0077	0.0035

PBS: population branch statistic. PBS′: the modified PBS. HNA: highland Native American. CLM: Colombians from Medellin, Colombia. CEU: Utah Residents with Northern and Western European Ancestry

[a] Chromosomal position in the middle of sliding window (NCBI build GRCh37)

Table S8. Three statistical values (weighted F_{ST}, PBS, and PBS′) in a ~436.3 kb genomic region containing *EGLN1* (a sliding window with 5 kb windows and 5 kb steps), calculated using the estimated allele frequency data of three populations (HNA, CLM, and JPT)

Position[a]	F_{ST} JPT-CLM	F_{ST} JPT-HNA	F_{ST} CLM-HNA	PBS_{JPT}	PBS_{CLM}	PBS_{HNA}	PBS'_{JPT}	PBS'_{CLM}	PBS'_{HNA}
1:231367500	0.0028	0.1342	0.1513	-0.0086	0.0114	0.1527	-0.0074	0.0098	0.1321
1:231372500	0.0111	0.0463	0.0199	0.0192	-0.0081	0.0282	0.0185	-0.0077	0.0271
1:231377500	-0.0077	0.0940	0.0793	0.0042	-0.0119	0.0945	0.0038	-0.0109	0.0870
1:231382500	0.0096	0.0487	0.0427	0.0080	0.0016	0.0420	0.0076	0.0015	0.0399
1:231387500	0.0125	0.0393	0.0573	-0.0031	0.0157	0.0433	-0.0030	0.0149	0.0410
1:231392500	0.0102	0.1188	0.1350	-0.0042	0.0144	0.1306	-0.0036	0.0127	0.1145
1:231397500	0.0170	0.0387	0.0683	-0.0070	0.0242	0.0465	-0.0066	0.0227	0.0437
1:231402500	-0.0032	0.0720	0.0960	-0.0147	0.0115	0.0894	-0.0135	0.0106	0.0823
1:231407500	0.0093	0.0283	0.0408	-0.0018	0.0111	0.0305	-0.0017	0.0107	0.0293
1:231412500	0.0077	0.0600	0.0631	0.0022	0.0055	0.0597	0.0020	0.0052	0.0559
1:231417500	0.0178	0.0505	0.0892	-0.0119	0.0298	0.0637	-0.0110	0.0275	0.0589
1:231422500	0.0072	0.0476	0.0459	0.0045	0.0027	0.0443	0.0043	0.0026	0.0421
1:231427500	0.0083	0.1180	0.0935	0.0179	-0.0095	0.1077	0.0160	-0.0085	0.0965
1:231432500	0.0213	0.0406	0.0177	0.0226	-0.0010	0.0189	0.0217	-0.0010	0.0181
1:231437500	0.0423	0.0372	0.0965	-0.0102	0.0534	0.0481	-0.0093	0.0489	0.0441
1:231442500	0.0346	0.1384	0.0744	0.0535	-0.0183	0.0955	0.0473	-0.0161	0.0845
1:231447500	0.0531	0.0193	0.0141	0.0299	0.0246	-0.0104	0.0287	0.0236	-0.0100
1:231452500	0.0164	0.0230	0.0220	0.0088	0.0078	0.0145	0.0085	0.0075	0.0140
1:231457500	-0.0003	0.1015	0.0871	0.0078	-0.0081	0.0992	0.0071	-0.0073	0.0903
1:231462500	0.0017	0.1309	0.1734	-0.0242	0.0259	0.1646	-0.0208	0.0222	0.1411
1:231467500	0.0001	0.1226	0.1410	-0.0106	0.0107	0.1413	-0.0093	0.0093	0.1238
1:231472500	-0.0006	0.1062	0.1205	-0.0083	0.0078	0.1206	-0.0075	0.0070	0.1077
1:231477500	0.0031	0.1118	0.1735	-0.0345	0.0375	0.1530	-0.0298	0.0325	0.1323
1:231482500	0.0037	0.0954	0.1325	-0.0191	0.0228	0.1194	-0.0170	0.0203	0.1063
1:231487500	0.0096	0.1232	0.1754	-0.0259	0.0355	0.1574	-0.0222	0.0304	0.1348
1:231492500	0.0032	0.1306	0.1767	-0.0256	0.0288	0.1656	-0.0219	0.0247	0.1417
1:231497500	-0.0041	0.1302	0.1626	-0.0210	0.0169	0.1606	-0.0182	0.0146	0.1388
1:231502500	-0.0011	0.1835	0.1987	-0.0099	0.0089	0.2126	-0.0082	0.0073	0.1755
1:231507500	0.0053	0.1542	0.1801	-0.0129	0.0182	0.1804	-0.0109	0.0154	0.1521
1:231512500	0.0011	0.1474	0.1934	-0.0272	0.0283	0.1867	-0.0229	0.0238	0.1572
1:231517500	-0.0022	0.1523	0.1922	-0.0252	0.0230	0.1904	-0.0212	0.0194	0.1603
1:231522500	-0.0017	0.1315	0.1644	-0.0202	0.0184	0.1612	-0.0174	0.0159	0.1390
1:231527500	0.0060	0.1365	0.1566	-0.0088	0.0148	0.1555	-0.0076	0.0128	0.1339
1:231532500	0.0007	0.1450	0.1855	-0.0239	0.0247	0.1806	-0.0203	0.0209	0.1528
1:231537500	0.0044	0.1435	0.2010	-0.0325	0.0369	0.1874	-0.0273	0.0310	0.1573
1:231542500	0.0622	0.1243	0.1841	-0.0033	0.0675	0.1360	-0.0027	0.0562	0.1133
1:231547500	0.0647	0.1953	0.2276	0.0130	0.0540	0.2043	0.0102	0.0424	0.1607
1:231552500	0.0748	0.0964	0.1511	0.0076	0.0701	0.0938	0.0065	0.0598	0.0800
1:231557500	0.0581	0.1575	0.0737	0.0773	-0.0175	0.0941	0.0670	-0.0151	0.0815
1:231562500	0.1020	0.3151	0.1632	0.1539	-0.0464	0.2245	0.1155	-0.0348	0.1685
1:231567500	0.0375	0.2291	0.0804	0.1073	-0.0691	0.1529	0.0901	-0.0580	0.1283
1:231572500	0.0572	0.1908	0.0700	0.0990	-0.0402	0.1127	0.0845	-0.0343	0.0962
1:231577500	0.1294	0.3791	0.1346	0.2353	-0.0967	0.2413	0.1705	-0.0701	0.1749
1:231582500	0.1007	0.3650	0.1340	0.2082	-0.1021	0.2460	0.1540	-0.0755	0.1819
1:231587500	0.0908	0.3708	0.2197	0.1552	-0.0600	0.3080	0.1106	-0.0427	0.2195
1:231592500	0.0875	0.3414	0.1754	0.1582	-0.0666	0.2594	0.1171	-0.0493	0.1920
1:231597500	0.0933	0.3445	0.1347	0.1878	-0.0899	0.2346	0.1409	-0.0675	0.1760
1:231602500	0.0488	0.1909	0.1107	0.0723	-0.0222	0.1395	0.0607	-0.0186	0.1173
1:231607500	0.0662	0.2009	0.0685	0.1109	-0.0424	0.1134	0.0938	-0.0359	0.0959
1:231612500	0.0218	0.3122	0.2012	0.0858	-0.0638	0.2885	0.0655	-0.0487	0.2201

1:231617500	0.0242	0.0353	0.0208	0.0197	0.0048	0.0162	0.0189	0.0046	0.0156
1:231622500	0.0017	0.0478	0.0870	-0.0202	0.0218	0.0692	-0.0188	0.0204	0.0646
1:231627500	0.1118	0.1879	0.0575	0.1337	-0.0152	0.0744	0.1121	-0.0127	0.0623
1:231632500	0.0433	0.2673	0.1468	0.0982	-0.0540	0.2127	0.0781	-0.0429	0.1692
1:231637500	0.0364	0.3064	0.1612	0.1136	-0.0765	0.2523	0.0881	-0.0593	0.1957
1:231642500	0.1020	0.1001	0.0142	0.0994	0.0082	0.0061	0.0892	0.0073	0.0055
1:231647500	0.0264	0.1722	0.0508	0.0818	-0.0551	0.1072	0.0722	-0.0485	0.0945
1:231652500	0.1029	0.2521	0.0735	0.1614	-0.0528	0.1292	0.1304	-0.0427	0.1044
1:231657500	0.0537	0.0834	0.0966	0.0203	0.0349	0.0667	0.0181	0.0311	0.0595
1:231662500	0.1229	0.2103	0.1097	0.1255	0.0056	0.1105	0.1011	0.0045	0.0890
1:231667500	0.0352	0.0758	0.0333	0.0404	-0.0045	0.0384	0.0376	-0.0042	0.0358
1:231672500	0.0500	0.2446	0.1476	0.0861	-0.0348	0.1945	0.0691	-0.0279	0.1561
1:231677500	0.0598	0.2844	0.0998	0.1456	-0.0839	0.1890	0.1164	-0.0671	0.1511
1:231682500	0.0661	0.1628	0.0941	0.0736	-0.0052	0.1040	0.0628	-0.0044	0.0887
1:231687500	0.0816	0.2123	0.0907	0.1143	-0.0292	0.1243	0.0945	-0.0242	0.1028
1:231692500	0.0572	0.3238	0.1279	0.1567	-0.0978	0.2346	0.1211	-0.0756	0.1814
1:231697500	0.0490	0.2166	0.1557	0.0626	-0.0123	0.1815	0.0508	-0.0100	0.1474
1:231702500	0.0787	0.1944	0.0810	0.1068	-0.0248	0.1094	0.0897	-0.0209	0.0918
1:231707500	0.0299	0.2061	0.1041	0.0756	-0.0452	0.1552	0.0638	-0.0381	0.1309
1:231712500	0.0214	0.1794	0.1173	0.0473	-0.0256	0.1504	0.0403	-0.0219	0.1283
1:231717500	0.0088	0.0740	0.0624	0.0107	-0.0018	0.0662	0.0099	-0.0017	0.0615
1:231722500	0.0490	0.1078	0.1375	0.0082	0.0420	0.1059	0.0071	0.0364	0.0916
1:231727500	0.0656	0.1219	0.0624	0.0667	0.0011	0.0633	0.0590	0.0010	0.0560
1:231732500	0.1830	0.0954	0.2194	0.0274	0.1748	0.0729	0.0215	0.1371	0.0572
1:231737500	0.2063	0.0518	0.2354	0.0079	0.2231	0.0453	0.0062	0.1748	0.0355
1:231742500	0.0839	0.0765	0.0381	0.0642	0.0235	0.0153	0.0582	0.0213	0.0139
1:231747500	0.2156	0.0929	0.0226	0.1587	0.0841	-0.0612	0.1343	0.0712	-0.0518
1:231752500	0.1433	0.0707	0.0343	0.0965	0.0581	-0.0232	0.0853	0.0514	-0.0205
1:231757500	0.1676	0.1074	0.0394	0.1284	0.0550	-0.0148	0.1099	0.0471	-0.0127
1:231762500	0.0990	0.0490	0.0169	0.0688	0.0355	-0.0185	0.0633	0.0327	-0.0170
1:231767500	0.1183	0.0600	0.0252	0.0811	0.0447	-0.0192	0.0733	0.0404	-0.0174
1:231772500	0.2024	0.0823	0.0179	0.1470	0.0792	-0.0611	0.1262	0.0680	-0.0525
1:231777500	0.1662	0.0763	0.0168	0.1221	0.0597	-0.0428	0.1072	0.0524	-0.0375
1:231782500	0.1026	0.0635	0.0106	0.0815	0.0267	-0.0160	0.0747	0.0244	-0.0146
1:231787500	0.0369	0.0541	0.1173	-0.0158	0.0534	0.0714	-0.0142	0.0482	0.0643
1:231792500	0.1251	0.0610	0.0224	0.0870	0.0467	-0.0240	0.0784	0.0421	-0.0217
1:231797500	0.0389	0.0347	0.0117	0.0316	0.0081	0.0037	0.0303	0.0077	0.0035

PBS: population branch statistic. PBS′: the modified PBS. HNA: highland Native American. CLM: Colombians from Medellin, Colombia. JPT: Japanese in Tokyo, Japan

[a] Chromosomal position in the middle of sliding window (NCBI build GRCh37)

Table S9. Three statistical values (weighted F_{ST}, PBS, and PBS′) in a ~436.3 kb genomic region containing *EGLN1* (a sliding window with 5 kb windows and 5 kb steps), calculated using the estimated allele frequency data of three populations (HNA, CLM, and YRI)

Position[a]	F_{ST} YRI-CLM	F_{ST} YRI-HNA	F_{ST} CLM-HNA	PBS_{YRI}	PBS_{CLM}	PBS_{HNA}	PBS'_{YRI}	PBS'_{CLM}	PBS'_{HNA}
1:231367500	0.0179	0.2287	0.1577	0.0531	-0.0350	0.2066	0.0433	-0.0286	0.1687
1:231372500	0.0376	0.0611	0.0049	0.0482	-0.0098	0.0148	0.0458	-0.0094	0.0140
1:231377500	0.0197	0.1447	0.0837	0.0443	-0.0245	0.1119	0.0392	-0.0216	0.0989
1:231382500	0.0198	0.0790	0.0582	0.0212	-0.0012	0.0612	0.0196	-0.0011	0.0566
1:231387500	0.0122	0.0985	0.0491	0.0328	-0.0205	0.0709	0.0303	-0.0190	0.0654
1:231392500	0.0147	0.1747	0.1403	0.0278	-0.0130	0.1642	0.0236	-0.0110	0.1393
1:231397500	0.0071	0.0856	0.0777	0.0079	-0.0008	0.0817	0.0072	-0.0007	0.0750
1:231402500	0.0069	0.1344	0.0932	0.0267	-0.0198	0.1176	0.0237	-0.0176	0.1046
1:231407500	0.0079	0.0925	0.0749	0.0136	-0.0057	0.0835	0.0124	-0.0052	0.0765
1:231412500	0.0202	0.1394	0.0497	0.0598	-0.0394	0.0903	0.0538	-0.0355	0.0813
1:231417500	0.0141	0.1106	0.0918	0.0176	-0.0034	0.0997	0.0158	-0.0030	0.0895
1:231422500	0.0508	0.0945	0.0313	0.0598	-0.0077	0.0395	0.0548	-0.0070	0.0362
1:231427500	0.0674	0.1724	0.1242	0.0632	0.0066	0.1260	0.0528	0.0055	0.1054
1:231432500	0.0839	0.1787	0.0189	0.1327	-0.0451	0.0641	0.1152	-0.0391	0.0556
1:231437500	0.0782	0.1549	0.0621	0.0928	-0.0114	0.0755	0.0802	-0.0099	0.0653
1:231442500	0.0601	0.1896	0.0760	0.0966	-0.0346	0.1136	0.0822	-0.0294	0.0967
1:231447500	0.0277	0.0592	0.0193	0.0348	-0.0067	0.0262	0.0330	-0.0063	0.0249
1:231452500	0.0459	0.1500	0.0248	0.0922	-0.0452	0.0703	0.0825	-0.0404	0.0629
1:231457500	0.0617	0.1548	0.1199	0.0521	0.0116	0.1161	0.0442	0.0098	0.0984
1:231462500	0.1145	0.1545	0.1883	0.0404	0.0812	0.1274	0.0323	0.0650	0.1020
1:231467500	0.0325	0.0676	0.1385	-0.0230	0.0561	0.0931	-0.0204	0.0498	0.0826
1:231472500	0.0432	0.0214	0.1288	-0.0360	0.0802	0.0576	-0.0327	0.0728	0.0523
1:231477500	0.0195	0.1630	0.2270	-0.0299	0.0496	0.2078	-0.0244	0.0404	0.1693
1:231482500	0.0206	0.0780	0.1322	-0.0198	0.0407	0.1011	-0.0177	0.0363	0.0901
1:231487500	0.0251	0.0932	0.1537	-0.0218	0.0472	0.1196	-0.0190	0.0412	0.1045
1:231492500	0.0444	0.0682	0.1815	-0.0421	0.0876	0.1128	-0.0364	0.0756	0.0974
1:231497500	0.0776	0.0219	0.1558	-0.0332	0.1140	0.0554	-0.0292	0.1003	0.0487
1:231502500	0.0433	0.1416	0.2490	-0.0447	0.0890	0.1974	-0.0360	0.0717	0.1590
1:231507500	0.0499	0.0614	0.1830	-0.0438	0.0949	0.1072	-0.0378	0.0820	0.0925
1:231512500	0.0697	0.0262	0.1739	-0.0461	0.1184	0.0727	-0.0403	0.1034	0.0635
1:231517500	0.0667	0.0112	0.1653	-0.0502	0.1193	0.0614	-0.0444	0.1055	0.0543
1:231522500	0.0519	0.0251	0.1570	-0.0461	0.0994	0.0715	-0.0410	0.0883	0.0635
1:231527500	0.0601	0.0171	0.1521	-0.0429	0.1049	0.0601	-0.0382	0.0935	0.0536
1:231532500	0.0494	0.0690	0.1934	-0.0463	0.0970	0.1179	-0.0397	0.0830	0.1009
1:231537500	0.0642	0.0303	0.1849	-0.0536	0.1200	0.0844	-0.0466	0.1043	0.0733
1:231542500	0.0261	0.1318	0.1863	-0.0192	0.0456	0.1606	-0.0162	0.0384	0.1353
1:231547500	0.0300	0.1508	0.1948	-0.0114	0.0418	0.1748	-0.0094	0.0347	0.1451
1:231552500	0.0369	0.1285	0.1880	-0.0166	0.0542	0.1541	-0.0139	0.0455	0.1293
1:231557500	0.0229	0.1326	0.0893	0.0359	-0.0128	0.1064	0.0318	-0.0113	0.0942
1:231562500	0.0442	0.2746	0.1850	0.0809	-0.0356	0.2401	0.0629	-0.0277	0.1868
1:231567500	0.0414	0.1500	0.1007	0.0494	-0.0071	0.1132	0.0427	-0.0061	0.0979
1:231572500	0.0237	0.0432	0.0882	-0.0121	0.0361	0.0562	-0.0112	0.0334	0.0520
1:231577500	0.0083	0.1356	0.1515	-0.0051	0.0134	0.1508	-0.0044	0.0116	0.1301
1:231582500	0.0104	0.1148	0.1362	-0.0070	0.0175	0.1290	-0.0061	0.0153	0.1132
1:231587500	0.0056	0.2091	0.2174	-0.0025	0.0081	0.2370	-0.0020	0.0065	0.1907
1:231592500	0.0038	0.1773	0.1989	-0.0114	0.0152	0.2065	-0.0094	0.0126	0.1706
1:231597500	0.0262	0.1678	0.1364	0.0319	-0.0053	0.1519	0.0270	-0.0045	0.1289
1:231602500	0.0142	0.1024	0.1114	0.0021	0.0122	0.1060	0.0019	0.0109	0.0946
1:231607500	0.0476	0.1119	0.0638	0.0507	-0.0020	0.0679	0.0454	-0.0018	0.0608
1:231612500	0.0425	0.1506	0.1196	0.0397	0.0038	0.1235	0.0340	0.0033	0.1058

1:231617500	0.0471	0.0917	0.0185	0.0629	-0.0146	0.0333	0.0581	-0.0135	0.0308
1:231622500	0.0195	0.0351	0.0602	-0.0033	0.0230	0.0390	-0.0031	0.0218	0.0369
1:231627500	0.1002	0.1985	0.0805	0.1215	-0.0159	0.0998	0.1008	-0.0132	0.0828
1:231632500	0.0418	0.2801	0.1365	0.1123	-0.0696	0.2164	0.0892	-0.0553	0.1719
1:231637500	0.0431	0.1980	0.1551	0.0481	-0.0040	0.1726	0.0395	-0.0033	0.1419
1:231642500	0.0305	0.0467	0.0205	0.0290	0.0019	0.0188	0.0277	0.0018	0.0179
1:231647500	0.0468	0.1275	0.0736	0.0539	-0.0060	0.0824	0.0477	-0.0053	0.0729
1:231652500	0.0329	0.1328	0.0785	0.0471	-0.0136	0.0954	0.0417	-0.0121	0.0845
1:231657500	0.0333	0.0521	0.0926	-0.0049	0.0388	0.0584	-0.0045	0.0355	0.0535
1:231662500	0.0479	0.1852	0.1666	0.0358	0.0133	0.1690	0.0294	0.0109	0.1387
1:231667500	0.0380	0.0435	0.0801	-0.0001	0.0389	0.0446	-0.0001	0.0359	0.0411
1:231672500	0.0498	0.1787	0.0654	0.0901	-0.0391	0.1067	0.0779	-0.0337	0.0922
1:231677500	0.0479	0.2304	0.1209	0.0911	-0.0420	0.1709	0.0747	-0.0344	0.1401
1:231682500	0.0463	0.1571	0.1419	0.0326	0.0148	0.1383	0.0275	0.0125	0.1166
1:231687500	0.0728	0.1798	0.1244	0.0705	0.0051	0.1278	0.0586	0.0042	0.1062
1:231692500	0.0484	0.2265	0.1325	0.0822	-0.0325	0.1747	0.0671	-0.0265	0.1427
1:231697500	0.0365	0.1242	0.1189	0.0216	0.0156	0.1110	0.0188	0.0136	0.0967
1:231702500	0.0525	0.1034	0.0885	0.0352	0.0187	0.0740	0.0312	0.0166	0.0656
1:231707500	0.0497	0.1692	0.1165	0.0562	-0.0053	0.1292	0.0476	-0.0045	0.1095
1:231712500	0.0821	0.1779	0.1110	0.0820	0.0037	0.1139	0.0684	0.0031	0.0950
1:231717500	0.0565	0.0621	0.0907	0.0136	0.0446	0.0505	0.0122	0.0402	0.0455
1:231722500	0.0816	0.0238	0.1610	-0.0332	0.1183	0.0573	-0.0291	0.1035	0.0502
1:231727500	0.0414	0.1164	0.0665	0.0486	-0.0063	0.0751	0.0435	-0.0057	0.0672
1:231732500	0.0899	0.1254	0.2200	-0.0102	0.1043	0.1442	-0.0082	0.0842	0.1164
1:231737500	0.0169	0.1474	0.2787	-0.0751	0.0921	0.2346	-0.0600	0.0736	0.1874
1:231742500	0.0254	0.0829	0.0756	0.0168	0.0090	0.0697	0.0153	0.0082	0.0636
1:231747500	0.0357	0.0432	0.0094	0.0355	0.0008	0.0087	0.0340	0.0008	0.0083
1:231752500	0.0368	0.0483	0.0344	0.0260	0.0115	0.0235	0.0245	0.0108	0.0221
1:231757500	0.0656	0.1069	0.0419	0.0690	-0.0012	0.0440	0.0621	-0.0011	0.0396
1:231762500	0.0923	0.1397	0.0266	0.1102	-0.0133	0.0403	0.0969	-0.0117	0.0354
1:231767500	0.0777	0.1260	0.0220	0.0966	-0.0158	0.0381	0.0864	-0.0141	0.0340
1:231772500	0.0395	0.1506	0.0041	0.0997	-0.0594	0.0635	0.0903	-0.0538	0.0575
1:231777500	0.0394	0.1042	0.0328	0.0584	-0.0183	0.0516	0.0535	-0.0167	0.0473
1:231782500	0.0658	0.0732	0.0099	0.0671	0.0010	0.0089	0.0623	0.0009	0.0083
1:231787500	0.0797	0.2680	0.1759	0.1008	-0.0177	0.2112	0.0779	-0.0137	0.1632
1:231792500	0.1060	0.1353	0.0206	0.1183	-0.0062	0.0271	0.1038	-0.0055	0.0237
1:231797500	0.1041	0.1593	0.0359	0.1234	-0.0135	0.0501	0.1064	-0.0116	0.0432

PBS: population branch statistic. PBS′: the modified PBS. HNA: highland Native American. CLM: Colombians from Medellin, Colombia. YRI: Yoruba in Ibadan, Nigeria
[a] Chromosomal position in the middle of sliding window (NCBI build GRCh37)

Table S10. PBS′ in a ~436.3 kb genomic region located 1 Mb upstream of *EGLN1* (a sliding window with 5 kb windows and 5 kb steps), calculated using the estimated allele frequency data for two population triplets (HNA, CLM, and JPT or CEU)

	CLM-HNA-JPT			CLM-HNA-CEU		
Position[a]	PBS'_{JPT}	PBS'_{CLM}	PBS'_{HNA}	PBS'_{CEU}	PBS'_{CLM}	PBS'_{HNA}
1:229932500	0.0462	-0.0375	0.2284	-0.0100	0.0109	0.1795
1:229937500	0.0745	-0.0631	0.3022	-0.0061	0.0023	0.2676
1:229942500	0.0285	-0.0268	0.2006	-0.0177	0.0214	0.1876
1:229947500	0.0405	-0.0346	0.2263	0.0069	-0.0084	0.2051
1:229952500	0.0294	-0.0319	0.3104	0.0054	-0.0054	0.2644
1:229957500	0.0436	-0.0341	0.2370	-0.0042	0.0010	0.2156
1:229962500	-0.0076	0.0128	0.0096	0.0156	-0.0093	0.0188
1:229967500	0.0333	-0.0313	0.2616	0.0262	-0.0289	0.2652
1:229972500	0.0457	-0.0407	0.2761	0.0222	-0.0231	0.2584
1:229977500	0.0361	-0.0395	0.3782	-0.0306	0.0289	0.3602
1:229982500	0.0499	-0.0447	0.2706	0.0242	-0.0230	0.2381
1:229987500	0.0002	0.0454	0.0082	-0.0092	0.0350	0.0133
1:229992500	0.0343	-0.0123	0.0388	0.0165	-0.0015	0.0515
1:229997500	0.0630	-0.0316	0.0657	0.0306	-0.0224	0.0615
1:230002500	0.0202	-0.0016	0.0268	0.0203	-0.0079	0.0434
1:230007500	0.0055	0.0209	0.0027	-0.0059	0.0185	0.0091
1:230012500	0.0029	0.0259	0.0176	0.0070	0.0124	0.0350
1:230017500	0.0083	0.0225	-0.0063	0.0081	-0.0008	0.0239
1:230022500	0.0959	-0.0432	0.0665	0.0054	-0.0053	0.0235
1:230027500	0.0011	0.0138	0.0111	0.0081	-0.0018	0.0127
1:230032500	0.0825	-0.0669	0.1530	0.0632	-0.0483	0.1771
1:230037500	0.0051	0.0337	0.0233	0.0093	-0.0128	0.0741
1:230042500	-0.0042	0.0223	0.0037	-0.0057	0.0009	0.0141
1:230047500	-0.0009	0.0166	-0.0049	-0.0057	0.0017	0.0159
1:230052500	-0.0038	0.0567	0.0316	-0.0190	0.0307	0.0726
1:230057500	-0.0090	0.0290	0.0071	-0.0124	0.0212	0.0185
1:230062500	0.0447	-0.0044	0.0303	-0.0124	0.0165	0.0284
1:230067500	-0.0005	0.0345	0.0104	-0.0208	0.0334	0.0359
1:230072500	-0.0001	0.0101	0.0079	0.0047	-0.0003	0.0195
1:230077500	0.0093	0.0023	0.0136	0.0015	0.0082	0.0258
1:230082500	0.0178	0.0034	0.0444	-0.0079	0.0042	0.0497
1:230087500	0.0029	-0.0024	0.0348	0.0019	-0.0021	0.0429
1:230092500	-0.0135	0.0082	0.0395	-0.0092	0.0052	0.0355
1:230097500	-0.0124	0.0117	0.0402	-0.0087	0.0094	0.0340
1:230102500	-0.0063	0.0051	0.0095	-0.0020	-0.0024	0.0276
1:230107500	0.0093	-0.0068	0.1213	0.0043	-0.0034	0.1422
1:230112500	-0.0054	0.0071	0.0700	0.0175	-0.0138	0.1088
1:230117500	-0.0015	0.0110	0.0181	0.0003	0.0041	0.0126
1:230122500	0.0219	-0.0045	0.0749	0.0358	-0.0291	0.1271
1:230127500	-0.0060	0.0178	0.0033	0.0108	-0.0121	0.0282
1:230132500	0.0162	-0.0004	0.0801	0.0000	-0.0009	0.0857
1:230137500	0.0377	-0.0050	0.0306	-0.0001	-0.0011	0.0280
1:230142500	0.0361	-0.0116	0.0348	-0.0018	-0.0026	0.0287
1:230147500	0.0166	0.0126	0.0297	-0.0041	0.0070	0.0413

1:230152500	-0.0010	0.0041	0.0366	-0.0066	0.0015	0.0282
1:230157500	0.0343	-0.0073	0.0257	0.0033	-0.0012	0.0219
1:230162500	0.0338	-0.0077	0.0211	0.0020	-0.0039	0.0348
1:230167500	0.0150	-0.0094	0.2114	0.0161	-0.0084	0.1994
1:230172500	0.0240	-0.0167	0.0331	0.0029	-0.0066	0.0203
1:230177500	0.0389	-0.0022	0.0223	0.0046	-0.0037	0.0220
1:230182500	0.0156	-0.0089	0.0533	-0.0006	-0.0014	0.0660
1:230187500	0.0352	-0.0162	0.0607	0.0076	-0.0057	0.0587
1:230192500	0.0315	-0.0213	0.0498	0.0003	-0.0028	0.0470
1:230197500	0.0377	-0.0072	0.0266	0.0074	-0.0037	0.0337
1:230202500	0.0570	0.0092	0.0003	0.0033	-0.0017	0.0084
1:230207500	0.0405	-0.0024	0.0097	0.0052	-0.0011	0.0062
1:230212500	0.0627	-0.0112	0.1104	0.0588	-0.0420	0.1339
1:230217500	0.0648	-0.0113	0.1280	0.0322	-0.0202	0.1588
1:230222500	0.0181	-0.0041	0.1056	0.0323	-0.0195	0.1286
1:230227500	0.0318	0.0175	0.0917	0.0009	-0.0031	0.1218
1:230232500	0.0408	-0.0149	0.0847	0.0115	-0.0118	0.0925
1:230237500	0.0280	-0.0190	0.1118	0.0130	-0.0172	0.1206
1:230242500	0.0724	-0.0399	0.1470	0.0138	-0.0167	0.1366
1:230247500	0.0387	-0.0223	0.0832	0.0009	-0.0067	0.0794
1:230252500	0.0354	-0.0256	0.1030	0.0130	-0.0158	0.1059
1:230257500	0.0754	-0.0078	0.0874	0.0224	-0.0206	0.0904
1:230262500	0.0272	0.0077	0.0813	0.0408	-0.0357	0.1200
1:230267500	0.0060	0.0156	0.0877	0.0275	-0.0164	0.1227
1:230272500	0.0241	-0.0081	0.0920	0.0402	-0.0264	0.1185
1:230277500	0.2312	-0.0193	0.0462	0.0307	-0.0210	0.0522
1:230282500	0.1961	0.0232	-0.0191	0.0037	-0.0026	0.0035
1:230287500	0.1668	0.0071	0.0104	-0.0022	-0.0017	0.0226
1:230292500	0.1560	0.0210	-0.0069	0.0056	-0.0062	0.0163
1:230297500	0.1798	0.0000	0.0226	0.0125	-0.0117	0.0252
1:230302500	0.1573	0.0130	0.0092	0.0083	-0.0068	0.0268
1:230307500	0.0926	0.0312	0.0235	0.0183	-0.0159	0.0683
1:230312500	0.1256	0.0171	0.0976	0.0383	-0.0263	0.1379
1:230317500	0.1402	0.0480	0.0706	0.0069	-0.0021	0.1262
1:230322500	0.0939	0.0408	0.0322	0.0065	-0.0036	0.0723
1:230327500	-0.0206	0.1114	0.1420	0.0590	-0.0450	0.2888
1:230332500	-0.0087	0.0675	0.2056	0.0790	-0.0541	0.3077
1:230337500	-0.0300	0.0794	0.0931	0.0610	-0.0464	0.2035
1:230342500	-0.0712	0.1279	0.1705	0.1191	-0.0942	0.3189
1:230347500	-0.0212	0.1196	0.1470	0.1413	-0.1092	0.3188
1:230352500	-0.0226	0.0858	0.1197	0.1012	-0.0777	0.2679
1:230357500	-0.0440	0.1889	0.0914	0.1262	-0.1007	0.3408

PBS′: the modified PBS. HNA: highland Native American. CLM: Colombians from Medellin, Colombia. JPT: Japanese in Tokyo, Japan. CEU: Utah Residents with Northern and Western European Ancestry

[a] Chromosomal position in the middle of sliding window (NCBI build GRCh37)

Table S11. PBS′ in a ~436.3 kb genomic region located 1 Mb downstream of *EGLN1* (a sliding window with 5 kb windows and 5 kb steps), calculated using the estimated allele frequency data for two population triplets (HNA, CLM, and JPT or CEU)

	CLM-HNA-JPT			CLM-HNA-CEU		
Position[a]	PBS'_{JPT}	PBS'_{CLM}	PBS'_{HNA}	PBS'_{CEU}	PBS'_{CLM}	PBS'_{HNA}
1:233742500	-0.0050	0.0274	0.0256	-0.0031	0.0029	0.0651
1:233747500	0.0013	0.0396	0.0274	0.0120	0.0004	0.0863
1:233752500	-0.0001	0.0186	0.0832	0.0139	0.0121	0.1289
1:233757500	0.0407	-0.0123	0.0323	0.0170	-0.0040	0.0321
1:233762500	0.0515	0.0009	0.0259	0.0522	-0.0118	0.0284
1:233767500	0.0589	0.0091	0.0209	0.0302	0.0009	0.0342
1:233772500	0.0392	-0.0156	0.1185	0.0555	-0.0376	0.1240
1:233777500	0.0316	-0.0165	0.0593	0.0409	-0.0186	0.0757
1:233782500	0.0392	-0.0282	0.0681	0.0297	-0.0076	0.0632
1:233787500	0.0152	-0.0122	0.0683	0.0359	-0.0117	0.0914
1:233792500	0.0186	-0.0069	0.0730	0.0180	0.0076	0.0750
1:233797500	0.0234	-0.0009	0.0143	0.0264	-0.0026	0.0254
1:233802500	0.0142	-0.0033	0.0184	0.0608	-0.0265	0.0467
1:233807500	0.0119	0.0116	0.0049	0.0346	0.0006	0.0183
1:233812500	0.0246	0.0027	0.0295	0.0289	-0.0134	0.0513
1:233817500	0.0332	0.0022	0.0114	0.0363	-0.0128	0.0347
1:233822500	0.0287	0.0072	0.0131	0.0198	0.0093	0.0154
1:233827500	0.0406	-0.0108	0.0176	0.0475	-0.0127	0.0286
1:233832500	0.0276	0.0118	0.0081	0.0060	0.0003	0.0247
1:233837500	0.0020	0.0083	0.0221	0.0705	-0.0056	0.0335
1:233842500	0.0144	0.0036	0.0180	0.0811	-0.0138	0.0463
1:233847500	0.0533	-0.0275	0.2327	0.0350	-0.0052	0.2836
1:233852500	0.0083	0.0053	0.0526	0.0771	-0.0278	0.0857
1:233857500	0.0187	0.0059	0.0323	0.0645	-0.0123	0.0527
1:233862500	-0.0023	0.0154	0.0378	0.0793	-0.0167	0.0783
1:233867500	0.0293	0.0116	0.0460	0.0148	0.0034	0.0573
1:233872500	0.0822	-0.0132	0.0589	0.0086	-0.0062	0.0556
1:233877500	0.0276	0.0455	0.0114	0.0437	-0.0179	0.0838
1:233882500	0.0420	0.0157	0.0163	0.0324	-0.0148	0.0561
1:233887500	0.0423	0.0131	0.0388	0.0348	-0.0042	0.0527
1:233892500	0.0461	0.0014	0.0266	0.0389	-0.0187	0.0553
1:233897500	0.0489	-0.0219	0.0729	0.0355	-0.0263	0.0731
1:233902500	0.0450	-0.0202	0.0872	0.0529	-0.0324	0.0903
1:233907500	0.0443	-0.0313	0.1082	0.0426	-0.0299	0.1214
1:233912500	0.0479	-0.0230	0.0792	0.0347	-0.0290	0.0950
1:233917500	-0.0041	-0.0006	0.0319	0.0126	-0.0112	0.0494
1:233922500	0.0207	-0.0090	0.0617	0.0289	-0.0160	0.0790
1:233927500	-0.0016	0.0795	0.0304	0.0268	-0.0021	0.1212
1:233932500	0.0351	0.0091	0.0302	0.0118	-0.0114	0.0662
1:233937500	0.0169	0.1043	-0.0108	0.0344	-0.0046	0.1045
1:233942500	0.0393	0.0651	0.0038	0.0363	-0.0129	0.0929
1:233947500	0.0220	0.0409	0.0111	0.0548	-0.0194	0.0800
1:233952500	0.0022	0.0269	0.0080	0.0729	-0.0187	0.0610
1:233957500	0.0125	0.0029	0.0179	0.0811	0.0049	0.0251

1:233962500	0.0094	-0.0047	0.0197	0.0893	-0.0034	0.0186
1:233967500	0.0013	-0.0004	0.0137	0.0955	0.0022	0.0158
1:233972500	0.0228	0.0017	0.0098	0.0731	0.0086	0.0140
1:233977500	0.0347	-0.0009	0.0229	0.0665	0.0019	0.0257
1:233982500	0.0343	0.0015	0.0233	0.0149	-0.0012	0.0447
1:233987500	0.0397	-0.0145	0.0359	0.0679	0.0028	0.0283
1:233992500	0.0647	-0.0427	0.0869	0.1434	-0.0619	0.1350
1:233997500	0.0564	-0.0232	0.0567	0.1511	-0.0407	0.0810
1:234002500	0.0801	-0.0380	0.0864	0.1720	-0.0357	0.0837
1:234007500	0.0722	-0.0341	0.0848	0.1373	0.0030	0.0405
1:234012500	0.0202	-0.0076	0.0165	0.0974	0.0125	0.0159
1:234017500	0.0425	-0.0031	0.0185	0.0752	-0.0067	0.0257
1:234022500	0.0059	0.0004	0.0278	0.1050	-0.0117	0.0422
1:234027500	0.0328	-0.0125	0.0303	0.0557	-0.0088	0.0250
1:234032500	0.0201	-0.0119	0.0618	0.1061	-0.0366	0.1030
1:234037500	0.0343	-0.0177	0.0803	0.1203	-0.0143	0.0791
1:234042500	0.0371	-0.0170	0.0945	0.1115	-0.0342	0.0949
1:234047500	0.0084	0.0255	0.0546	0.0642	-0.0182	0.0965
1:234052500	-0.0025	0.0099	0.1075	0.0621	-0.0247	0.1565
1:234057500	0.0110	-0.0121	0.2027	0.0525	-0.0082	0.2090
1:234062500	-0.0092	0.0059	0.1325	0.0505	-0.0093	0.1498
1:234067500	0.0068	-0.0106	0.1418	0.0763	-0.0264	0.1530
1:234072500	-0.0057	0.0104	0.1374	0.0527	-0.0110	0.1658
1:234077500	0.0271	-0.0182	0.1170	0.0523	-0.0198	0.1564
1:234082500	0.0308	-0.0258	0.0997	0.0594	-0.0249	0.1111
1:234087500	0.0147	-0.0047	0.0091	0.0700	-0.0295	0.0388
1:234092500	-0.0006	0.0111	0.0157	0.0128	-0.0071	0.0256
1:234097500	-0.0142	0.0394	0.0383	0.0009	0.0047	0.0870
1:234102500	-0.0186	0.0467	0.0358	0.0005	0.0070	0.0779
1:234107500	0.0098	0.0199	-0.0053	0.0151	-0.0043	0.0167
1:234112500	0.0058	0.0210	0.0036	0.0294	-0.0011	0.0386
1:234117500	-0.0025	0.0257	0.0391	0.0195	-0.0077	0.0740
1:234122500	0.0146	0.0252	0.0201	0.0200	-0.0108	0.0739
1:234127500	0.0215	0.0589	-0.0121	0.0154	-0.0094	0.0658
1:234132500	-0.0006	0.0764	0.0075	0.0105	-0.0068	0.0967
1:234137500	-0.0030	0.0722	0.0380	0.0376	-0.0007	0.1205
1:234142500	-0.0271	0.0639	0.0637	0.0593	-0.0377	0.1522
1:234147500	-0.0055	0.0598	0.0073	0.0276	-0.0138	0.1016
1:234152500	0.0055	0.0125	0.0305	0.0588	-0.0391	0.0969
1:234157500	0.0001	0.0001	0.0315	0.0262	-0.0123	0.0639
1:234162500	-0.0239	0.0468	0.0585	0.0883	-0.0386	0.1358
1:234167500	-0.0267	0.0454	0.0567	0.0409	-0.0272	0.1350

PBS′: the modified PBS. HNA: highland Native American. CLM: Colombians from Medellin, Colombia. JPT: Japanese in Tokyo, Japan. CEU: Utah Residents with Northern and Western European Ancestry

[a] Chromosomal position in the middle of sliding window (NCBI build GRCh37)
