# Supplementary material for: Effect of EGLN1 Genetic Polymorphisms on Hemoglobin Concentration in Andean Highlanders
Source: Biomed Res Int. 2020 Nov 15;2020:3436581. doi: 10.1155/2020/3436581 (PMC7686849; doi:10.1155/2020/3436581)
Supplement: Supplementary 2 — Supplement Figure S1: the physical position and linkage disequilibrium of four SNPs across an ~81 kb genomic region around EGLN1 in 99 Bolivian highlanders. Supplement Figure S2: the estimated hemoglobin (Hb) concentrations (g/dL) in individuals with different EGLN1 rs1769792 genotypes in La Paz, El Alto, and the entire cohort. Supplement Figure S3: relationship between the estimated hemoglobin (Hb) concentration (g/dL) and EGLN1 haplotype frequency in the Bolivian cohorts. Supplement Figure S4: haplotype bifurcation plots for an ~200 kb genomic region containing EGLN1. Supplement Figure S5: genetic structure analysis of 342 individuals from BLV, PEL, CLM, MXL, CEU, JPT, and YRI populations. [file 3436581.f2.pdf]

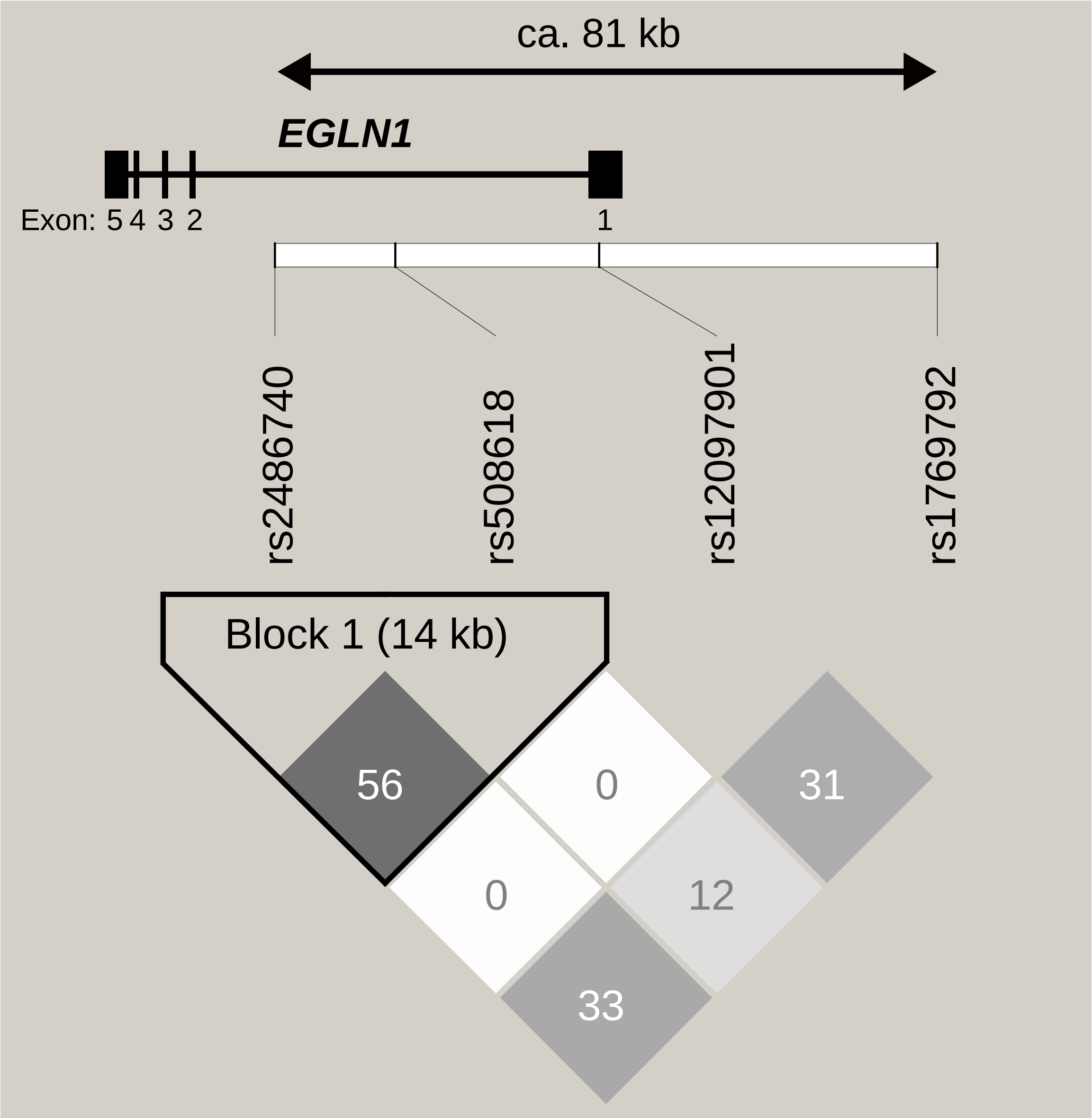

Figure S1. The physical position and linkage disequilibrium of four SNPs across a ~81 kb genomic region around *EGLN1* in 99 Bolivian highlanders. The diagram was created using Haploview ver. 4.2. Numbers in diamonds represent r^2 values (×100).

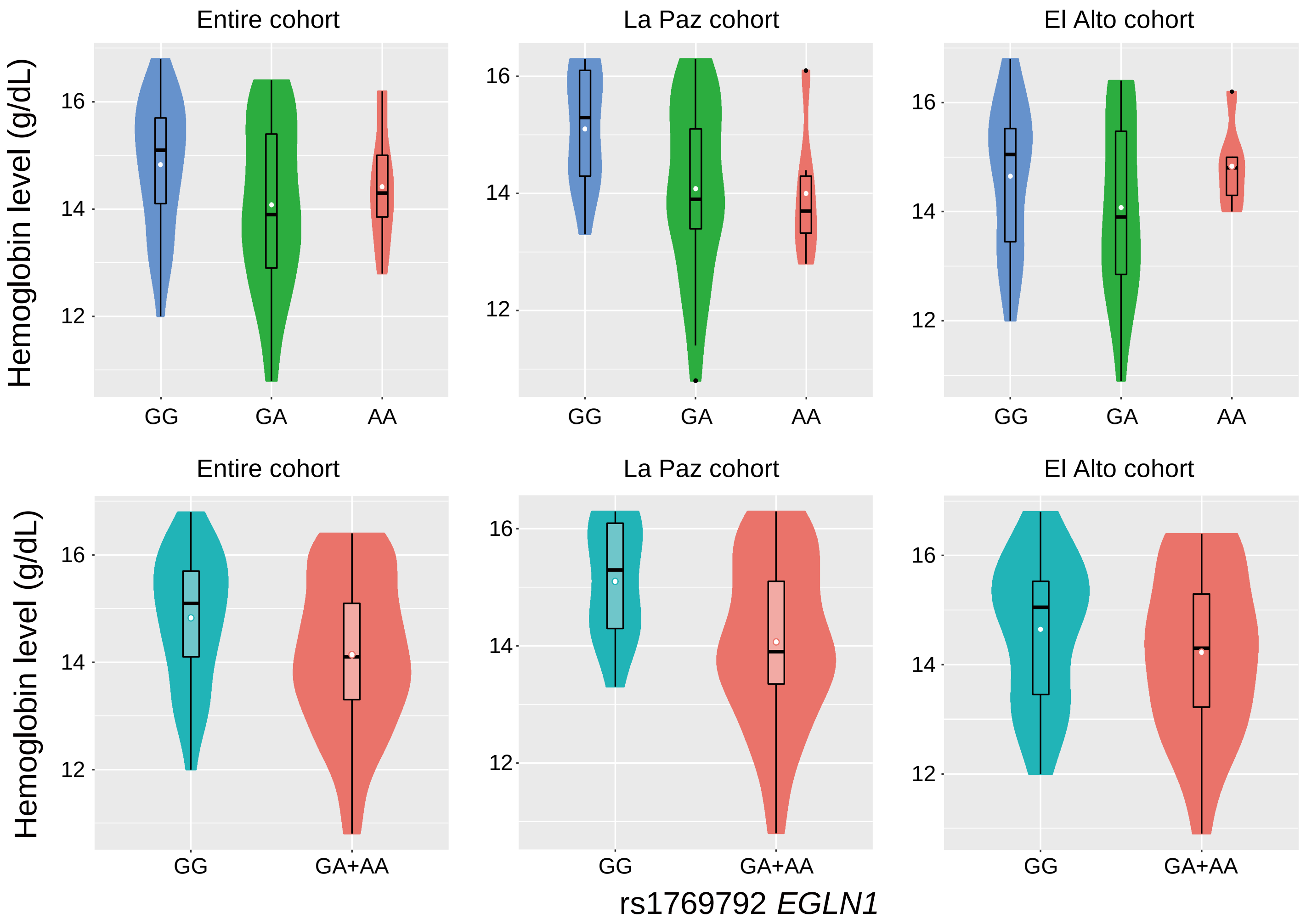

Figure S2. The estimated hemoglobin (Hb) concentrations (g/dL) in individuals with different *EGLN1* rs1769792 genotypes in La Paz, El Alto, and the entire cohort. White dots represent mean Hb concentration values and bold black bars represent median values.

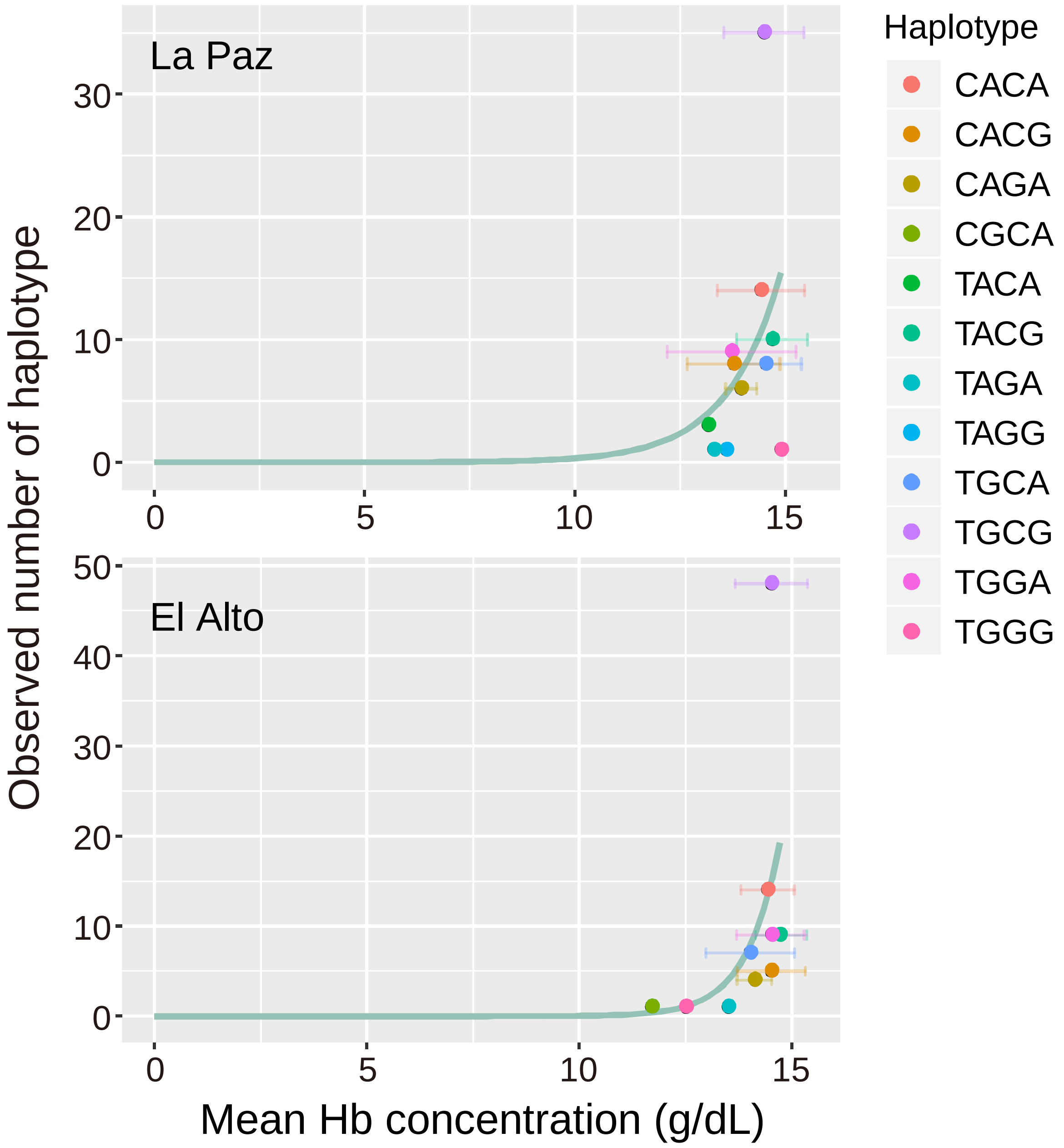

Figure S3. Relationship between the estimated hemoglobin (Hb) concentration (g/dL) and *EGLN1* haplotype frequency in the Bolivian cohorts. Hb levels are represented as the mean ± standard deviation of the mean. The bold blue line represents the regression line. Different colored circles indicate different haplotypes estimated from four *EGLN1* SNPs (rs2486740, rs508618, rs12097901, and rs1769792).

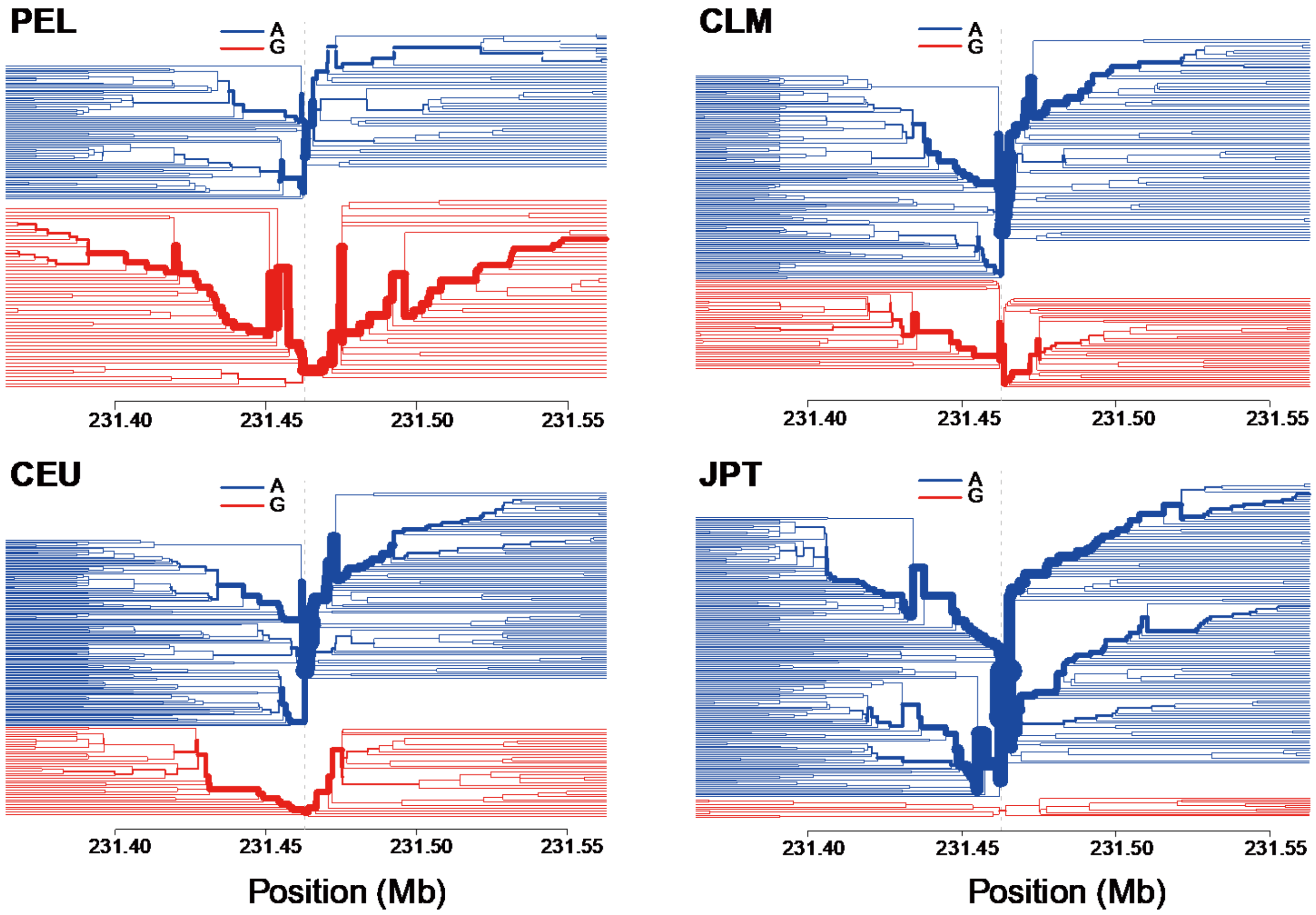

Figure S4. Haplotype bifurcation plots for a ~200 kb genomic region containing *EGLN1*. Vertical dashed line represents the position of the focal marker (rs1769792). The horizontal axis represents chromosomal position (NCBI build GRCh38). Line thickness represents the number of chromosomes with a haplotype. PEL: Peruvians from Lima, Peru. CLM: Colombians from Medellin, Colombia. CEU: Utah Residents with Northern and Western European Ancestry. JPT: Japanese in Tokyo, Japan.

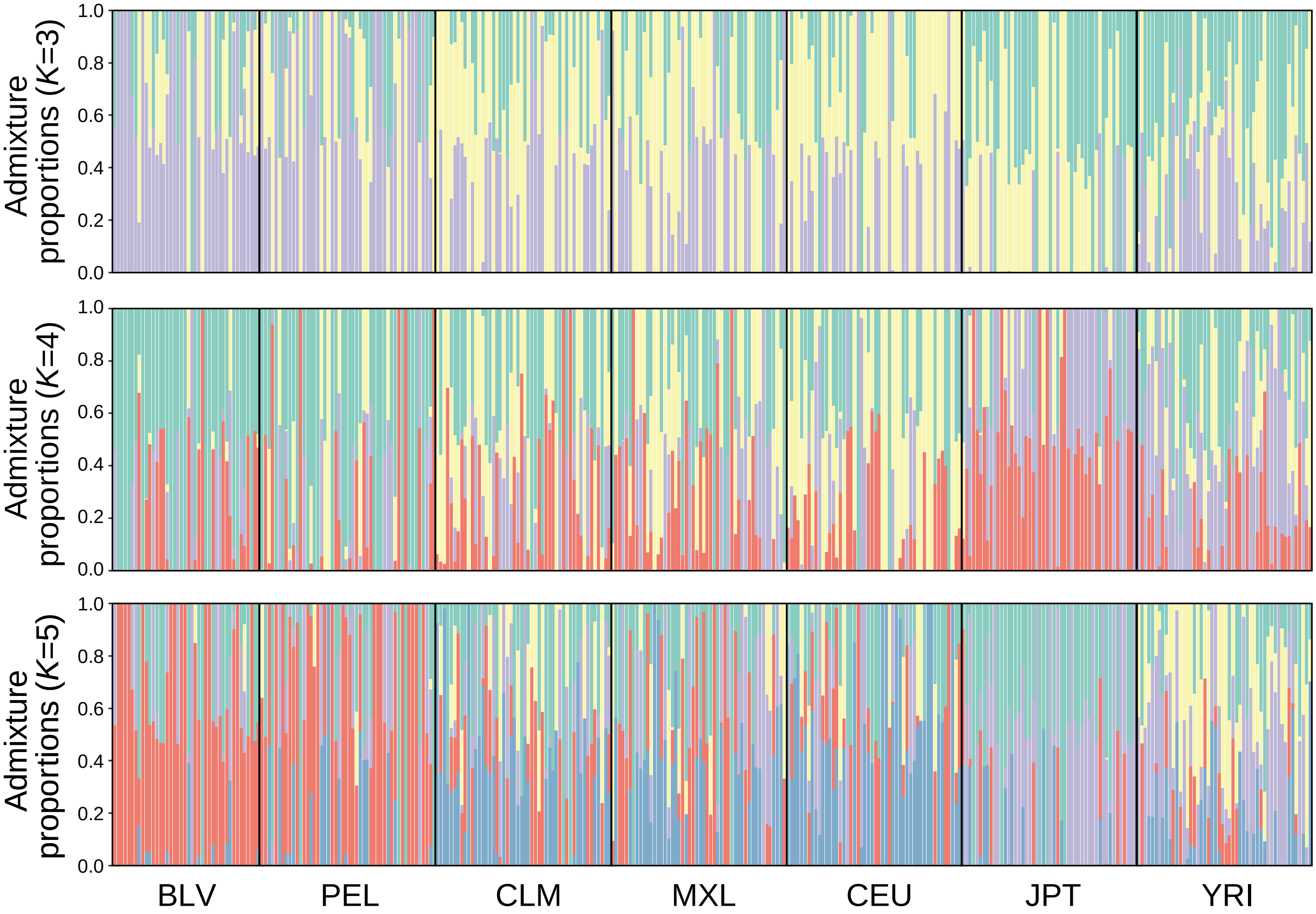

Figure S5. Genetic structure analysis of 342 individuals from BLV, PEL, CLM, MXL, CEU, JPT, and YRI populations. *K* indicates the number of putative ancestral populations. Colors represent proportions of genetic ancestry from the assumed ancestral population (admixture proportions). BLV: Bolivian from La Paz and El Alto, Bolivia. PEL: Peruvians from Lima, Peru. CLM: Colombians from Medellin, Colombia. MXL: Mexican Ancestry from Los Angeles USA. CEU: Utah Residents with Northern and Western European Ancestry. JPT: Japanese in Tokyo, Japan. YRI: Yoruba in Ibadan, Nigeria.
